# The tumor-associated fibroblasts regulate urothelial carcinoma progression

**DOI:** 10.1093/jmcb/mjaf032

**Published:** 2025-09-19

**Authors:** Yu Xiao, Junfeng Yang, Mengjie Sun, Yongfu Li, Qinyin Liu, Jinjun Leng, Maolin Yang, Jinrui Wang, Hongju Li, Caifeng Yang, Changfen Luo, Jiahong Li, Longli Kang, Fen Huang, Yanhong Yu, Chuanmao Zhang

**Affiliations:** The Academy for Cell and Life Health, Faculty of Life Science and Technology and Medical School, Kunming University of Science and Technology, and The First People’s Hospital of Yunnan Province/The Affiliated Hospital Department of Urology, Kunming University of Science and Technology, Kunming 650032, China; The Academy for Cell and Life Health, Faculty of Life Science and Technology and Medical School, Kunming University of Science and Technology, and The First People’s Hospital of Yunnan Province/The Affiliated Hospital Department of Urology, Kunming University of Science and Technology, Kunming 650032, China; The Academy for Cell and Life Health, Faculty of Life Science and Technology and Medical School, Kunming University of Science and Technology, and The First People’s Hospital of Yunnan Province/The Affiliated Hospital Department of Urology, Kunming University of Science and Technology, Kunming 650032, China; The Key Laboratory of Cell Proliferation and Differentiation of the Ministry of Education, College of Life Sciences, Peking University, Beijing 100871, China; The Academy for Cell and Life Health, Faculty of Life Science and Technology and Medical School, Kunming University of Science and Technology, and The First People’s Hospital of Yunnan Province/The Affiliated Hospital Department of Urology, Kunming University of Science and Technology, Kunming 650032, China; Fujian Provincial Key Laboratory of Tumor Biotherapy, Clinical Oncology School of Fujian Medical University, Fujian Cancer Hospital, Fuzhou 350014, China; The Academy for Cell and Life Health, Faculty of Life Science and Technology and Medical School, Kunming University of Science and Technology, and The First People’s Hospital of Yunnan Province/The Affiliated Hospital Department of Urology, Kunming University of Science and Technology, Kunming 650032, China; The Academy for Cell and Life Health, Faculty of Life Science and Technology and Medical School, Kunming University of Science and Technology, and The First People’s Hospital of Yunnan Province/The Affiliated Hospital Department of Urology, Kunming University of Science and Technology, Kunming 650032, China; The Academy for Cell and Life Health, Faculty of Life Science and Technology and Medical School, Kunming University of Science and Technology, and The First People’s Hospital of Yunnan Province/The Affiliated Hospital Department of Urology, Kunming University of Science and Technology, Kunming 650032, China; The Academy for Cell and Life Health, Faculty of Life Science and Technology and Medical School, Kunming University of Science and Technology, and The First People’s Hospital of Yunnan Province/The Affiliated Hospital Department of Urology, Kunming University of Science and Technology, Kunming 650032, China; The Academy for Cell and Life Health, Faculty of Life Science and Technology and Medical School, Kunming University of Science and Technology, and The First People’s Hospital of Yunnan Province/The Affiliated Hospital Department of Urology, Kunming University of Science and Technology, Kunming 650032, China; The Academy for Cell and Life Health, Faculty of Life Science and Technology and Medical School, Kunming University of Science and Technology, and The First People’s Hospital of Yunnan Province/The Affiliated Hospital Department of Urology, Kunming University of Science and Technology, Kunming 650032, China; The Academy for Cell and Life Health, Faculty of Life Science and Technology and Medical School, Kunming University of Science and Technology, and The First People’s Hospital of Yunnan Province/The Affiliated Hospital Department of Urology, Kunming University of Science and Technology, Kunming 650032, China; The Academy for Cell and Life Health, Faculty of Life Science and Technology and Medical School, Kunming University of Science and Technology, and The First People’s Hospital of Yunnan Province/The Affiliated Hospital Department of Urology, Kunming University of Science and Technology, Kunming 650032, China; Key Laboratory for Molecular Genetic Mechanisms and Intervention Research on High Altitude Disease of Tibet Autonomous region, Key Laboratory of High-Altitude Environment and Genes Related to Diseases of Tibet Autonomous Region, School of Medicine, Xizang Minzu University, Xianyang 712082, China; The Academy for Cell and Life Health, Faculty of Life Science and Technology and Medical School, Kunming University of Science and Technology, and The First People’s Hospital of Yunnan Province/The Affiliated Hospital Department of Urology, Kunming University of Science and Technology, Kunming 650032, China; The Academy for Cell and Life Health, Faculty of Life Science and Technology and Medical School, Kunming University of Science and Technology, and The First People’s Hospital of Yunnan Province/The Affiliated Hospital Department of Urology, Kunming University of Science and Technology, Kunming 650032, China; The Academy for Cell and Life Health, Faculty of Life Science and Technology and Medical School, Kunming University of Science and Technology, and The First People’s Hospital of Yunnan Province/The Affiliated Hospital Department of Urology, Kunming University of Science and Technology, Kunming 650032, China; The Key Laboratory of Cell Proliferation and Differentiation of the Ministry of Education, College of Life Sciences, Peking University, Beijing 100871, China

**Keywords:** upper tract urothelial carcinoma, cancer-associated fibroblasts, multi-omics research, tumor microenvironment

## Abstract

Tumor-associated fibroblasts (CAFs) regulate tumorigenesis, tumor cell proliferation, and metastasis via secreting related regulatory factors; however, the role of CAFs in regulating the development of upper tract urothelial carcinoma (UTUC) remains unclear. Here, by utilizing single-cell RNA sequencing, single-nucleus RNA sequencing, SpaTial enhanced resolution omics-sequencing, and UTUC immunofluorescence chip cohort to construct the first comprehensive microenvironmental atlas of CAFs, we investigated the roles of CAFs in UTUC progression. Through hierarchical clustering and the copy number variation scores of UTUC epithelial cells, we classified tumor epithelial cells into high-malignant, medium-malignant, and low-malignant potential categories. We found pronounced interaction signals between different CAF subclusters and all three types of epithelial cells, among which high-malignant epithelial cells exhibited the most significant communication signals with the myofibroblastic CAFs1 (myCAFs1) and myCAFs2 subclusters, and FN1 and COL1A1 generated by CAFs played critical roles in this process, suggesting that the progression of UTUC may be attributed to the activation of tumor cells by CAFs. Additionally, both myCAFs1 and myCAFs2 simultaneously affect bladder urothelial carcinoma (BUC) prognosis, with the risk model showing good consistency across cohorts. Therefore, this study constructs a multi-omics landscape of UTUC and identifies common prognostic markers shared with BUC.

## Introduction

Urothelial carcinoma (UC) is a common urinary cancer that can develop in various parts of the urinary system, including the renal pelvis, ureter, bladder, and urethra. Despite the different morphological structures and histological manifestations between upper tract UC (UTUC) and bladder UC (BUC), the clinical diagnosis and treatment for UTUC is mainly based on the research focused on BUC (Hassler et al., 2020). BUC is the most prevalent form of UC, accounting for 90%–95%, with ∼75% being non-muscle invasive bladder cancer (Kamat et al., 2016). In contrast, UTUC originates from the upper urinary tract and typically exhibits greater invasiveness and a worse prognosis. More than half of the patients with UTUC are initially diagnosed with muscle-invasive UC, and UTUC accounts for ∼5%–10% of all UC cases (Margulis et al., 2009; Siegel et al., 2021). The lack of specific research evidence pertaining to UTUC limits our understanding of this cancer type and constrains our ability to develop more effective treatments. Consequently, it is essential that further research should be conducted specifically on UTUC to enhance understanding, diagnosis, and therapeutic strategies for UTUC.

As immune sentinel cells, fibroblasts affect human health and have the ability to activate and regulate immune responses to mediate disease progression. During the process of tumor formation, fibroblasts are activated (Driskell et al., 2013; Arina et al., 2016). At the initial stage of activation, fibroblasts can promote the body’s resistance to tumor progression. Interestingly, when a tumor occurs, cancer cells seem to be able to ‘hijack’ the defense mechanism of fibroblasts to support tumor growth (Buechler et al., 2021; Herrera et al., 2021). CAFs can secrete CXCL12 and VEGFA to promote endothelial angiogenesis and secrete factors such as LIF, HGF, and IGF1 that can interact with tumor cells to induce tumor growth and metastasis (Shi et al., 2019). CAFs play an important role in BUC. Recently, a new subcluster of CAFs with high expression of SLC14A1 from BUC was identified, which was found significantly enriched in patients tending to have poor prognosis and chemotherapeutic drug resistance (Ma et al., 2022). Single-cell RNA sequencing (scRNA-seq) has been used to reveal the tumor immune microenvironment in UTUC (Liang et al., 2022). However, the omics study of scRNA-seq alone might obscure the abundance of stromal cells (Lake et al., 2017; Wen et al., 2022).

In this work, by integrating scRNA-seq, single-nucleus RNA sequencing (snRNA-seq), and SpaTial enhanced resolution omics-sequencing (Stereo-seq) from tumor tissues and adjacent non-tumor tissues, which enables high-resolution, high-throughput, single-cell level observation of UTUC (Chen et al., 2022), we analyzed the UTUC microenvironmental characteristics. Further validation was conducted by using multiplex immunofluorescence chip (MIC) cohorts consisting of 120 UTUC tissues, 50 para-cancer tissues, and 25 paired BUC and para-cancer tissues, as well as multiple UC cohorts (TCGA-BLCA, GSE13507, and IMvigor210). Collectively, our data have effectively confirmed the mutual relationship between UTUC and BUC, providing a new insight into the stromal microenvironment in UC.

## Results

### The study designs

In this study, we utilized a multi-cohort, multi-omics integrated analysis. The cohorts we generated are the scRNA-seq, snRNA-seq, Stereo-seq, and MIC cohorts. The public database cohorts include the TCGA-BLCA transcriptome and mutation cohorts, GSE13507 cohort, and IMvigor210 cohort.

### UTUC cell type identification

**Figure 1:**
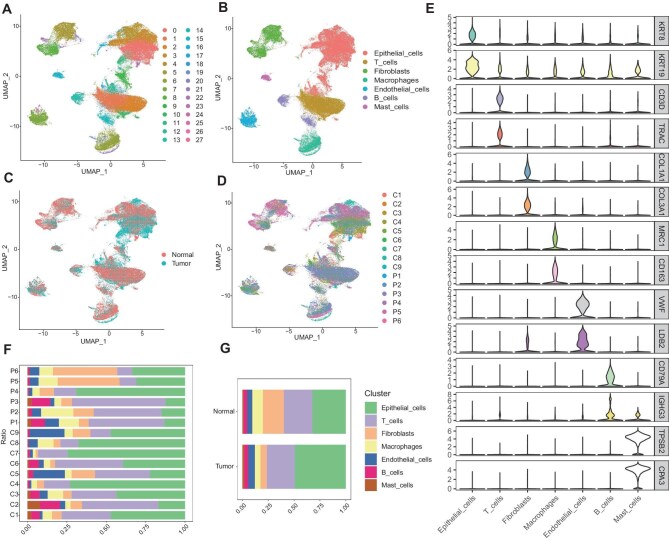
UTUC single-cell atlas. (**A**) A total of 136042 cells were clustered into 28 distinct cell clusters. (**B**) Seven UTUC cell types were identified. (**C**) Group by tissues. (**D**) Group by samples. C, cancer sample; P, para-cancer sample. (**E**) Violin plot displaying the expression of marker genes specific to UTUC cell types. (**F** and **G**) The proportion of each cell type in the samples.

Through merging of the scRNA-seq and snRNA-seq data followed by quality control analysis, we retained 136042 high-quality single cells, which were then clustered into 28 distinct cell clusters. Based on known marker genes, we identified seven cell types (Figure 1A–E): epithelial cells (KRT8, KRT19), T cells (CD3D, TRAC), fibroblasts (COL1A1, COL3A1), macrophages (MRC1, CD163), endothelial cells (LDB2, VMF), B cells (IGHG3, CD79A), and mast cells (TPSB2, CPA3). Epithelial cells constitute a significant proportion in both tumor and normal tissues, with a higher proportion observed in tumor tissues (Figure 1F and G). Notably, there are also differences in the abundance of immune cells between tumor and normal tissues. These findings provide initial evidence of the differential characteristics and heterogeneity of UTUC.

### Difference analysis

After conducting differential analysis between tumor and normal tissues, a total of 5587 differentially expressed genes (DEGs) were identified by the cut-off of adjusted *P*-value <0.05 and absolute log2(fold change) (|log2FC|) >0.5. Among them, 1473 genes exhibited |log2FC| > 1. The classification ‘Highly’ is represented as |log2FC| > 1, ‘Up’ as |log2FC| > 0, and the rest as ‘Lowly’ and ‘Down’. The cell types are represented as 0–6, corresponding to epithelial cells, T cells, fibroblasts, macrophages, endothelial cells, B cells, and mast cells (Supplementary Figure S1A). Additionally, the five most significant differential genes were marked in each cell type. Furthermore, gene set variation analysis (GSVA) was performed on epithelial cells to show the differences between tumor epithelium and normal epithelium (Supplementary Figure S1B). Through the comparison of metabolic pathways between tumor and normal tissues, we observed significant metabolic changes in UTUC (Supplementary Figure S1C and D). Notably, the metabolism of tumor epithelial cells is more active, particularly in pathways related to fatty acid metabolism, lipid metabolism, and others.

### UTUC epithelial landscape

**Figure 2:**
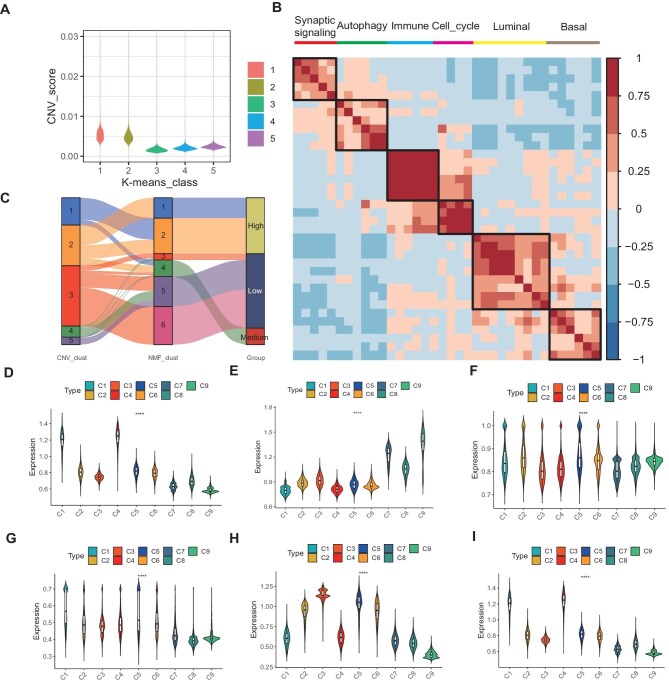
UTUC co-expressed module characteristics. (**A**) The tumor epithelial cells were re-clustered into five k-means classes, and the CNV scores were calculated for each k-means class. (**B**) Hierarchical clustering was utilized to assess the correlation among six major metaprograms in nine tumor tissue samples. The results are visually represented as a heatmap, with labels above the metaprograms denoting their main features. (**C**) The Sankey diagram illustrates the associations between different groups, ultimately categorizing the six distinct modules into three categories: HMECs, MMECs, and LMECs. (**D**–**I**) Signature scores of six major metaprograms in nine cancer samples and ranked in order.

We assessed the malignancy level of epithelial cells. K-means unsupervised clustering (*n* = 5) was employed and the copy number variation (CNV) scores were calculated for each class (Figure 2A). To identify co-expressed modules that were distinct in multiple tumors, we employed NMF analysis. Hierarchical clustering enabled us to identify six main distinct modules, each representing a unique intratumoral heterogeneity signature, associated with functions related to synaptic signaling transmission, autophagy regulation, immune response activation, the cell cycle, the luminal isoform, and the basal isoform, respectively (Figure 2B). Gene Ontology (GO) enrichment analysis was performed to reveal the pathway characteristics of these six modules (Supplementary Figure S2A). Since Modules 1 and 2 primarily correspond to the K-means classes with high CNV scores (1 and 2 in Figure 2A), Modules 3, 5, and 6 correspond to those with low CNV scores (3, 4, and 5 in Figure 2A), and Module 4 corresponds to both, we define Modules 1 and 2 as high-malignant epithelial cells (HMECs), Module 4 as medium-malignant epithelial cells (MMECs), and Modules 3, 5, and 6 as low-malignant epithelial cells (LMECs) (Figure 2C). We extracted the top 30 signature genes of each module and found distinct signature scores among cancer samples, highlighting the complex intratumoral characteristics of UTUC (Figure 2D–I). This information suggests the potential to stratify patients based on signature genes. Furthermore, to investigate the prognostic impact of 180 signature genes from the six NMF modules on BUC, we conducted a differential analysis comparing tumor and normal tissues using the TCGA-BLCA cohort (Supplementary Figure S2B). The expression levels of the intersection genes in TCGA-BLCA were extracted. Univariate and multivariate Cox regression analyses identified five potential prognostic genes, including LGALS3, BRINP3, PTPRR, XIST, and S100A16 (Supplementary Figure S2C–E).

### Trajectory analysis and transcription factor characteristics of epithelial cells

We utilized monocle analysis to carry out data quality control and explore potential differential characteristics of malignant epithelial cells. Remarkably, we identified a low-to-medium-to-high pattern in malignant epithelial cells (Supplementary Figure S3A). We also examined the variation in the expression of the top 9 quantitative genes in UTUC epithelial cells (Supplementary Figure S3B). The branched expression analysis model (BEAM) revealed distinct expression patterns among epithelial cell branches, further illuminating the inherent differential characteristics of epithelial cells during the development of UTUC and providing evidence for the evolution of its malignant degree (Supplementary Figure S3D). In addition, we investigated the pathway characteristics of different malignant epithelial cells by GSVA (Supplementary Figure S3C). The characteristics of transcription factors in malignant epithelial cells were analyzed by pySCENIC. The top 5 most significant transcription factors of each NMF module were extracted and visualized in a heatmap, providing further evidence of heterogeneity among the six modules (Supplementary Figure S4).

### CAF subclusters of UTUC

**Figure 3:**
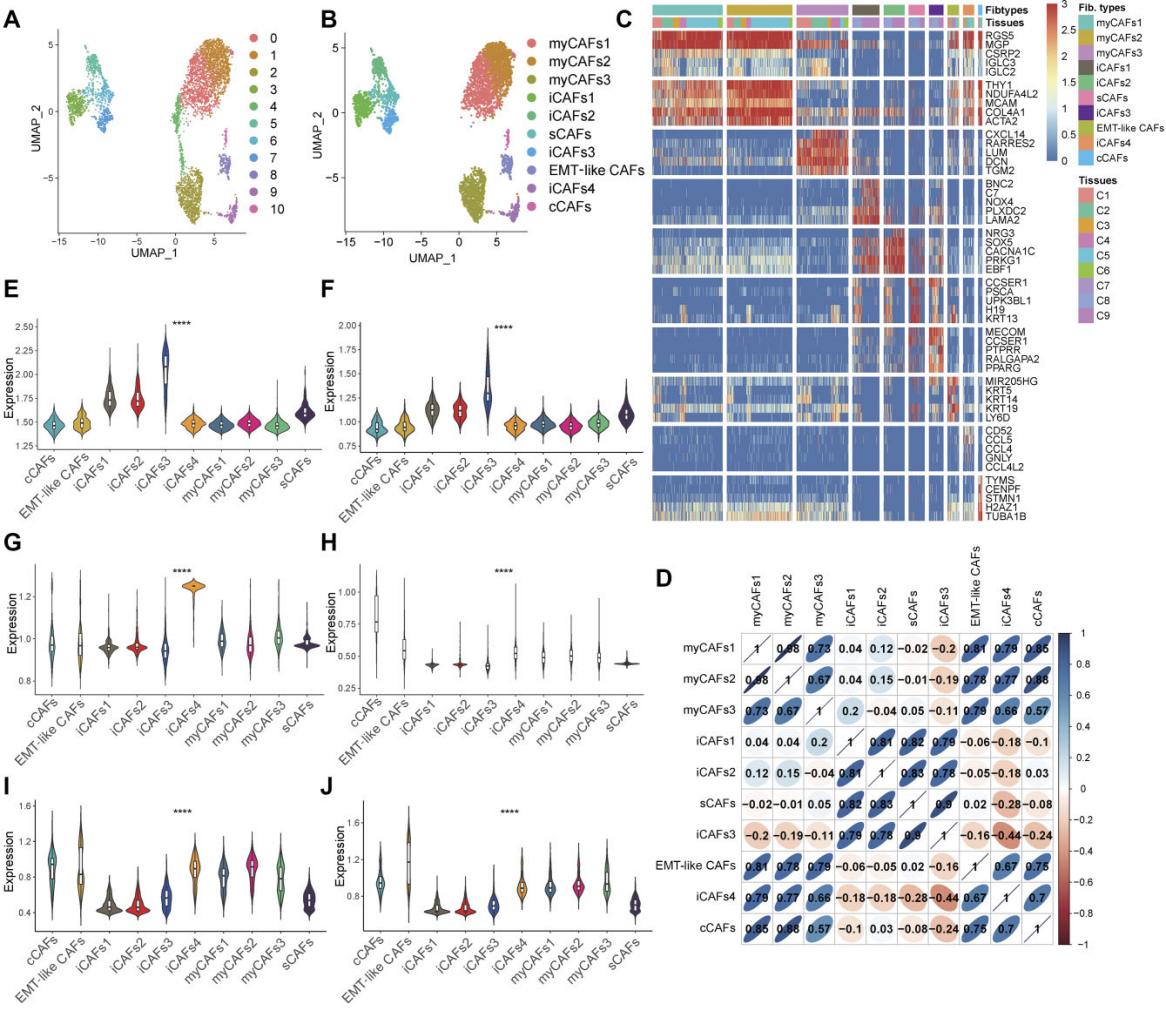
CAF subcluster characteristics. (**A** and **B**) Fibroblasts were re-clustered into 11 subclusters. After removing Cluster 4, which highly expresses a ribosome-related gene, the fibroblasts were annotated into 10 subclusters. (**C**) The heatmap of marker genes differentially expressed in each CAF subcluster across nine UTUC tissue samples. The selected genes are highlighted, and the color gradient on the heatmap represents their respective expression levels. (**D**) The correlation heatmap illustrates the relationships among CAF subclusters in UTUC. The numbers indicate the correlations between different subclusters, with color coded to represent the strength of the correlation. (**E**–**J**) Signature scores of six major metaprograms in CAF subclusters and ranked in order.

Tumor formation is accompanied by fibroblast activation, and the extracellular matrix formation of tumor cells is highly influenced by CAFs. CAFs exert their influence on tumor growth and prognosis through collagen production, cytokine secretion, angiogenesis induction, and mediation of immune escape (Sahai et al., 2020; Chen et al., 2021). Currently, no study has been conducted on the single-cell level of UTUC fibroblasts, which could be crucial for enhancing the prognosis of UTUC and BUC. In this study, CAFs from UTUC tissues were isolated and re-clustered into 11 subclusters (Figure 3A). Cluster 4 was excluded due to a ribosome-related gene as the primary marker, and subsequently the subclusters were re-labeled as myofibroblastic CAFs1 (myCAFs1), myCAFs2, myCAFs3, inflammatory CAFs1 (iCAFs1), iCAFs2, stem CAFs (sCAFs), iCAFs3, EMT-like CAFs, iCAFs4, and cycle CAFs (cCAFs), respectively (Figure 3B and C). The pathway characteristics of each CAF subcluster are annotated using GO terms (Supplementary Figure S5). Spearman correlation analysis was employed to investigate the correlations between different CAF subclusters (Figure 3D). Additionally, we evaluated the single-sample gene set enrichment analysis (ssGSEA) scores of the top 30 genes of each NMF module in CAF subclusters and found significantly different expression patterns of different modules among CAF subclusters (Figure 3E–J).

### Interactions between CAFs and epithelial cells

**Figure 4:**
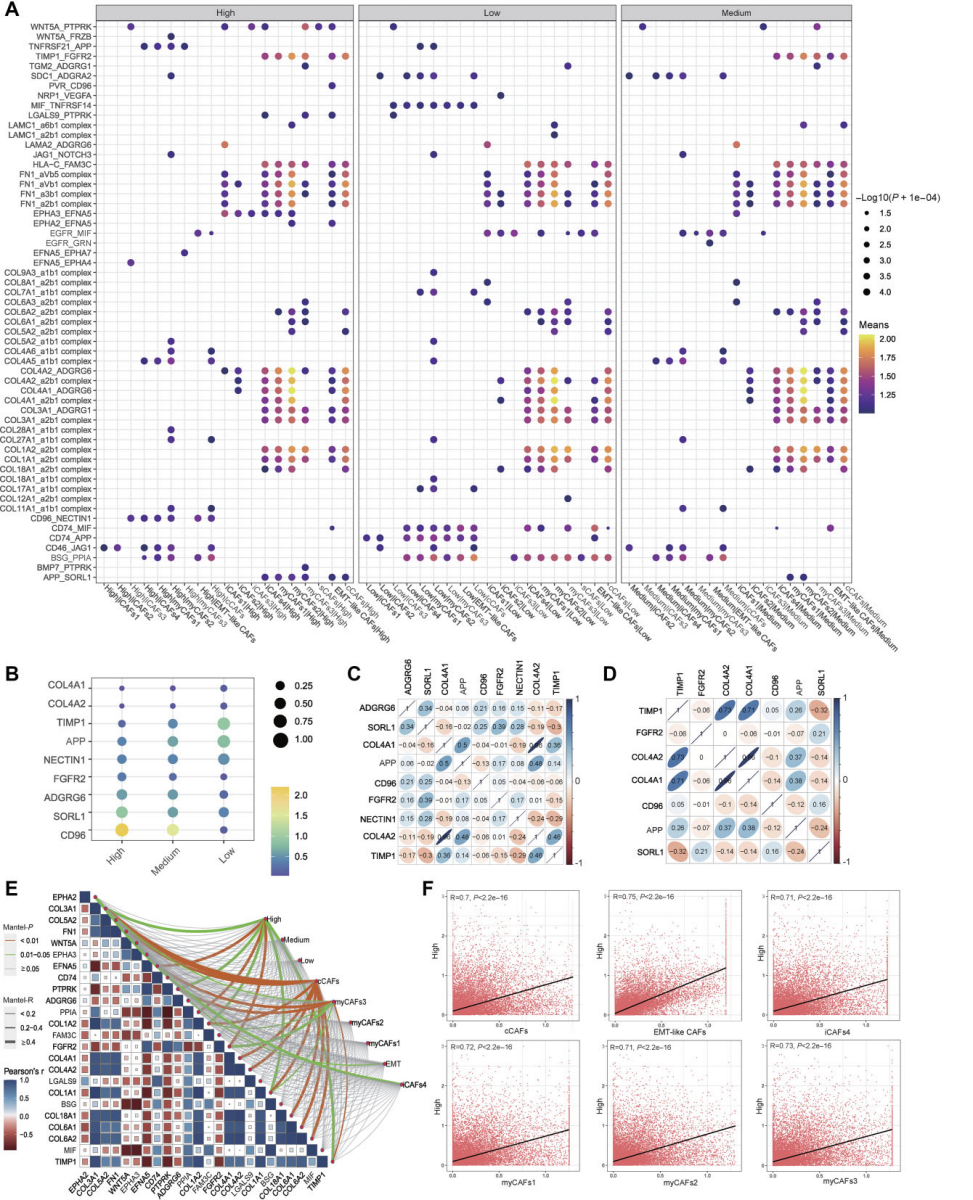
CellPhoneDB analysis of UTUC epithelial cells and CAFs. (**A**) The bubble plot displays the most significant interactions between the three types of UTUC epithelial cells and CAFs. The left legend corresponds to the receptor–ligand pair, where the size of each point reflects the *P*-value, and the color represents the expression value of the receptor–ligand pair. The filtering criteria include *P*-value < 0.05 and mean-value > 1. (**B**) The bubble plot of nine genes exhibiting significant expression changes in three types of UTUC epithelial cells. (**C** and **D**) Correlation analysis of nine genes with the TCGA-BLCA cohort and the IMvigor210 cohort. (**E**) The Mantel test was employed to examine the correlations between the most significantly expressed genes of receptor–ligand pairs and the indicated nine cell types. The curve color indicates the *P*-value, while the thickness reflects the Mantel correlation. The box color and size represent the intergenic Spearman correlation. (**F**) Spearman correlation analysis between HMECs and six CAF subclusters.

By utilizing CellPhoneDB, we investigated the interaction characteristics between CAFs and epithelial cells in the context of UTUC. Our analysis revealed significant communication features between different CAF subclusters (cCAFs, EMT-like CAFs, myCAFs3, myCAFs2, myCAFs1, and iCAFs4) and all three types of epithelial cells (Figure 4A). Notably, nine genes (TIMP1, FGFR2, COL4A2, ADGRG6, COL4A1, CD96, NECTIN1, APP, and SORL1) exhibited evident expression changes in LMECs, MMECs, and HMECs (Figure 4B). We then performed a correlation analysis of this group of genes with the TCGA-BLCA cohort and the IMvigor210 cohort (Figure 4C and D). Remarkably, both cohorts displayed similar characteristics, suggesting that CAFs were equally effective in patients with BUC, regardless of whether they received immunotherapy or not. Moreover, a stronger correlation was observed between HMECs and these identified genes (Figure 4E). The correlation scatterplot results demonstrated a significant association between epithelial cells exhibiting close communication and CAFs (Figure 4F; Supplementary Figure S6).

### Establishment of the CAF-related risk model

**Figure 5:**
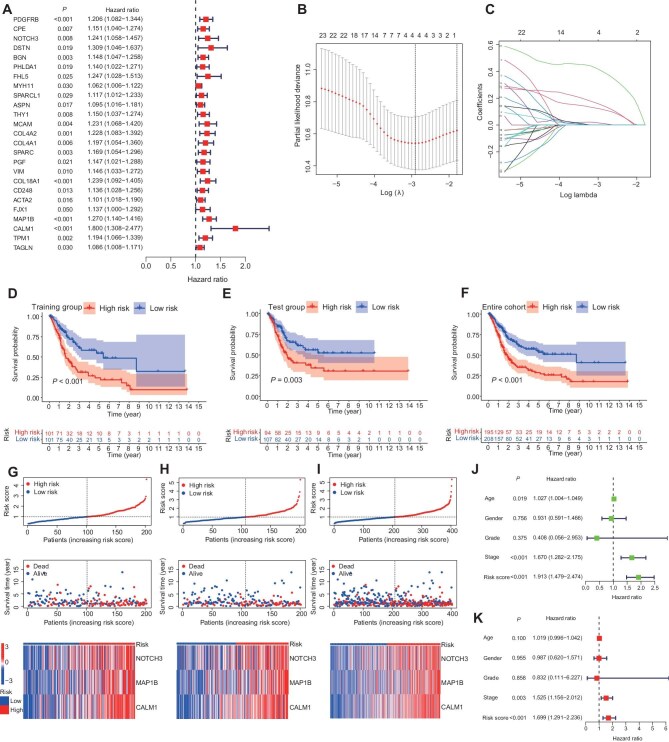
Prognostic models predict patient outcomes. (**A**) Univariate Cox regression analysis of myCAFs2 and myCAFs1 marker gene expression levels in the TCGA-BLCA cohort. (**B** and **C**) Cross-validation results of parameter selection in the Lasso regression model. (**D**–**F**) The survival analysis results for the training group, the test group, and the entire cohort, with color coded to indicate high- and low-risk patients. (**G**–**I**) The survival distribution of patients in the training group, the test group, and the entire cohort based on the risk model, showing a positive correlation, i.e. as the risk score increases, the number of patient deaths and the expression level of risk genes also increase. (**J** and **K**) Univariate and multivariate Cox regression analyses demonstrate the optimal performance for risk score.

To construct a CAF-related risk model for clinical prediction in BUC, we extracted the top 30 marker genes of each CAF subcluster to calculate ssGSEA scores in the TCGA-BLCA and IMvigor210 cohorts. The samples were then divided into high- and low-score groups. Survival analysis was performed in the TCGA-BLCA cohort, and immunotherapy responses were analyzed in the IMvigor210 cohort (Supplementary Figure S7). The results for myCAFs2 and myCAFs1 were most favorable. In addition, myCAFs2 and myCAFs1 exhibit the most significant communication with malignant epithelial cells. Hence, we used myCAFs1 and myCAFs2 for risk model construction. The expression levels of the top 30 marker genes of myCAFs1 and myCAFs2 were extracted for univariate Cox regression analysis (Figure 5A). Interestingly, myCAFs1 and myCAFs2 were found in the terminal differentiation stage of pseudotime, and the gene expression levels positively correlated with pseudotime (Figure 5A; Supplementary Figure S8). Subsequently, we randomly split the TCGA-BLCA cohort into a training group and a test group, with an equal number of participants in each group. The training group was subjected to Lasso Cox regression to further refine the selected factors, resulting in the construction of a multivariate Cox regression model (Figure 5B and C). Ultimately, three genes were identified for the development of risk models (CALM1, NOTCH3, and MAP1B). The survival analysis demonstrated that this risk model effectively predicted the clinical outcome of UC, with high-risk patients experiencing a worse prognosis (Figure 5D). The test group and entire cohort serving as the internal validation groups confirmed the ability of this risk model to predict clinical outcomes in BUC patients (Figure 5E and F). In the three groups of BUC patients, we observed a positive correlation between the risk score, the risk of death, and the gene expression level (Figure 5G–I). In addition, the risk model could independently predict the prognosis of BUC patients (Figure 5J and K).

### External validation of the risk model

Further evaluation of the immunotherapy response in BUC patients provided additional evidence for the effectiveness of the risk model, showing that patients in the low-risk group were more likely to benefit from immunotherapy (Supplementary Figure S9A). For external validation, survival analysis was performed in the GSE13507 and IMvigor210 cohorts, showing significant differences between low- and high-risk groups (Supplementary Figure S9B–D). The risk score also successfully predicted immunotherapy response in BUC patients within the IMvigor210 cohort (Supplementary Figure S9E). Additionally, a preliminary validation of three risk model genes by immunohistochemistry demonstrated different staining distributions in BUC and normal tissues (Supplementary Figure S9F; https://www.proteinatlas.org/).

### Significant mutation types in BUC

A recent large-scale mutational genomics study revealed genetic mutation characteristics in UTUC (Fujii et al., 2021). Here, we examined the significant mutational genes in BUC and found that TP53 mutations comprised the majority, succeeded by FGFR3 mutations, with RAS mutations representing a smaller fraction (Supplementary Figure S10A). Consistent expression patterns were observed across all three bulk cohorts. In the high-risk group, the FN1 and COL gene families exhibited notably high expression levels (Supplementary Figure S10B–D) and displayed inverse correlations with immune checkpoints, potentially contributing to poor prognosis (Supplementary Figure S10E). Furthermore, in an intricate network of protein interactions among these genes, the Betweenness Centrality of each gene was assessed, and the top 10 genes were identified, among which FN1 shows the utmost degree of Betweenness Centrality (Supplementary Figure S10F). Enrichment analysis subsequently illuminated that the top 10 genes predominantly influence key biological processes in BUC associated with cell proliferation, apoptosis, energy metabolism, and the cell cycle (Supplementary Figure S10G).

### Validation of FN1, COL1A1, and CALM1 in UTUC and BUC MIC cohorts

**Figure 6:**
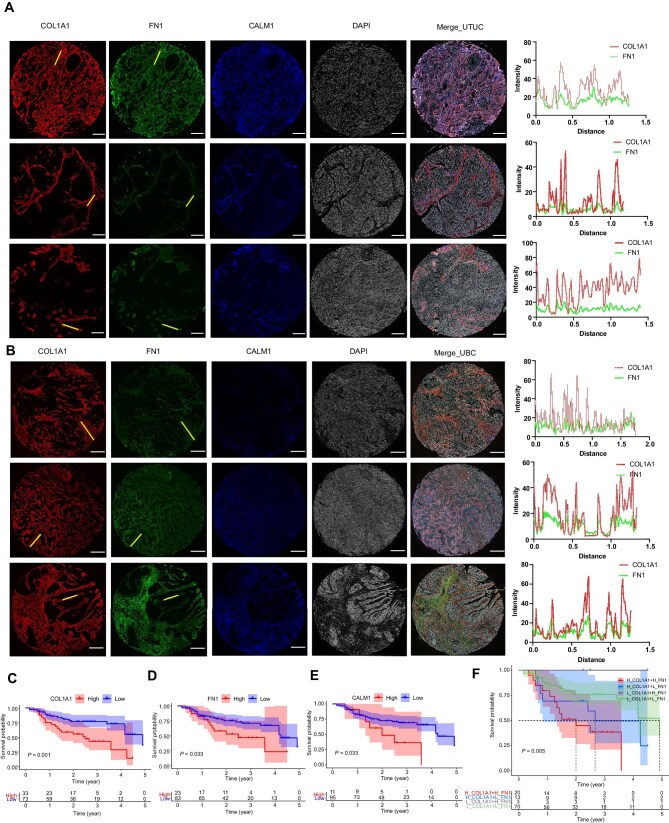
Validation of FN1, COL1A1, and CALM1 in the MIC cohorts. (**A** and **B**) Multiplex immunofluorescence staining of three UTUC tissue samples (**A**, scale bar, 200 μm) and three BUC tissue samples (**B**, scale bar, 400 μm). The images demonstrate the localization characteristics of FN1, COL1A1, and CALM1, as well as the co-localization of FN1 and COL1A1, by measuring the fluorescence intensity at the yellow lines. (**C**–**F**) The impact of different expression levels of FN1, COL1A1, and CALM1 on the 5-year survival rate of UTUC. The results indicate that FN1, COL1A1, and CALM1 are high-risk predictive factors for UTUC.

Subsequently, FN1, COL1A1, and CALM1, which had the highest risk coefficient, were validated using MIC cohorts. The Plot Profile function of ImageJ was used to measure the grayscale values of FN1 and COL1A1 at the yellow lines in the MICs, revealing evident co-localization of FN1 and COL1A1 in both UTUC and BUC (Figure 6A and B). The 5-year survival analyses revealed that UTUC patients with high expression levels of FN1, COL1A1, and CALM1 had poorer prognoses (Figure 6C–F), consistent with the results for BUC patients. Furthermore, differential expression analysis in both UTUC and BUC paired with corresponding para-cancer tissues showed significant differences in the expression of FN1, COL1A1, and CALM1 (Supplementary Figure S11A and B). High expression levels of FN1, COL1A1, and CALM1 were positively correlated with poorer clinicopathological characteristics (Supplementary Figure S11C).

### Characteristics of CAFs in Stereo-seq

Using snRNA-seq and scRNA-seq data as references, we identified major cellular components on the UTUC slides and performed spatial annotation through SPOTlight, in which CAF subclusters were merged, i.e. myCAFs1, myCAFs2, and myCAFs3 were combined into myCAFs, and dotted lines indicate the tumor margin area (Supplementary Figure S12A–D). We also investigated the spatial expression patterns of FN1, COL1A1, and CALM1 and observed that FN1 and COL1A1 were mainly localized at the tumor cell margins and exhibited co-localization, with Spearman correlation analysis showing a positive correlation (Supplementary Figures S12E and 13A–C). Similarly, myCAFs showed positively correlated expression with three types of malignant epithelial cells (Supplementary Figure S13F–H), and the pseudotime trajectory characteristics of CAF subclusters and each type of malignant epithelial cells are shown at the spatial level (Supplementary Figure S13D and E). To further investigate the impact of myCAFs on tumors, we analyzed the immune microenvironmental characteristics of UTUC (Supplementary Figure S14A–F). Notably, tumor cell regions within the barrier structure formed by FN1 and COL1A1 were enriched with immunosuppressive cell subpopulations, such as CD4^+^TOX^+^ Treg cells and M2-type macrophages (CD163^+^Macro and LRMDA^+^Macro), while HLA^+^ B cells, which initiate immune responses, were isolated outside the barrier (Supplementary Figure S14G). This suggests that the physical barrier formed by FN1 and COL1A1 suppresses immune cell function and promotes tumor progression.

## Discussion

As a rare subtype of UC, UTUC lacks high-level research evidence to guide clinical treatment. In this study, utilizing multi-omics method for the first time, we present a comprehensive analysis of the interaction between fibroblasts and tumor cells in UTUC. By exploring the molecular characteristics of UTUC at a high resolution on the cellular level, we identified potential prognostic markers for BUC. Specifically, we identified myCAFs1 and myCAFs2 as predictors of prognosis and immunotherapy response among CAF subclusters significantly interacting with tumor cells. After multiple rounds of screening, we ultimately selected NOTCH3, MAP1B, and CALM1 to construct the risk model. Previous studies often inferred the biological characteristics of UTUC by exploring BUC, which obscures the true biological behavior of UTUC, thereby impacting the poor prognosis outcomes for patients to some extent. We aim to find common ground while acknowledging differences, seeking consistency with BUC through our investigation of UTUC, ultimately providing novel evidence to inform clinical decision-making.

Our study revealed close communication between the myCAFs1 and myCAFs2 subclusters and tumor cells, establishing their effectiveness in predicting prognostic outcomes and UC immunotherapy response. In the context of pancreatic ductal adenocarcinoma, CAFs play a significant role in the active interaction of cancer cells within the tumor microenvironment (Hosein et al., 2020). Notably, different subclusters of CAFs exhibit distinct biological characteristics, with the myCAF and iCAF subclusters potentially influencing the progression of pancreatic cancer. In prostate cancer, the presence of SPP1^+^ myCAFs was found to confer resistance to androgen deprivation therapy through an SPP1–ERK paracrine mechanism, thereby associating the myCAF subclusters with poorer treatment outcomes (Wang et al., 2023). We discovered that CAF-mediated tumor development operates by influencing the stability of the extracellular matrix through FN1 and COL gene families, and a notable correlation exists between these factors. These families may play a role in the transformation of normal fibroblasts into CAFs, influencing extracellular matrix remodeling, and establishing a reciprocal feedback loop with tumor cells to drive tumor advancement. Previous studies demonstrated that CAFs can sequester tumor cells, thereby impeding contact between malignant cells and cytotoxic immune cells to facilitate immune escape (Luo et al., 2022; Jia et al., 2025). Here, we observed that FN1 and COL1A1 derived from myCAFs form a physical barrier, which obstructs immune-mediated tumor cell killing and promotes the enrichment of immunosuppressive cell subclusters, ultimately driving tumor progression.

The research on immunotherapy has significantly revolutionized the treatment landscape of modern medicine, yielding promising outcomes for numerous tumor patients. However, for advanced cancers, failed targeted therapy or immunotherapy can lead to patient mortality. Our constructed model accurately predicts the overall survival and progression-free survival of UC patients, and its effectiveness was duly validated in the independent validation set. In the TCGA-BLCA cohort, the risk model demonstrates its ability to predict the efficacy of immunotherapy in patients, while in the IMvigor210 cohort, it successfully predicts the overall survival and immunotherapy response of BUC patients after immunotherapy, with both cohorts showing improved prognoses and immune efficacy in the low-risk group. Cancer stem cells are deemed pivotal drivers of tumorigenesis, and Notch signaling plays a role in tumor initiation by entering ligand-expressing cells through endocytosis. Upregulation and activation of Notch signaling are mechanisms contributing to tumor hormone resistance, chemotherapy resistance, and immunotherapy resistance (Kopan and Ilagan, 2009; Sansone et al., 2016; Aster et al., 2017). In triple-negative breast cancer, the expression level of NOTCH3 is significantly increased (Park et al., 2010). Moreover, NOTCH3 has been implicated in the recurrence of ovarian cancer (Wang et al., 2015). Previous studies also identified MAP1B as a high-risk gene in BUC, and its expression level negatively correlates with the prognosis of BUC (Gu et al., 2022; Tan et al., 2023). CALM proteins, a class of highly conserved calcium receptors, include CALM1, which plays crucial roles in cell proliferation, differentiation, and motility signaling pathways (Wang et al., 2018; Lei et al., 2021). CALM1 expression has shown significant correlations with these prostate and bladder cancers in urological tumors (Adeola et al., 2016; Zhang et al., 2018). Importantly, FN1 and COL1A1 expression positively correlated with these risk-associated genes, suggesting potential synergistic interactions. Notably, high-risk patients exhibited immune drug-resistance traits, a phenomenon potentially linked to the FN1- and COL1A1-mediated physical barrier that impedes targeted agent cytotoxicity against tumor cells. Collectively, this study delineates CAFs within the UC microenvironment, identifying specific subclusters that govern prognosis and progression in UC. Our discovery of the FN1- and COL1A1-constituted physical barrier as a promoter of urothelial carcinogenesis provides a framework for developing personalized therapeutic strategies.

While our findings reveal the prognostic value of CAFs in UC, we acknowledge limitations, including the absence of a prospective UTUC cohort for validation and comparative analysis. Furthermore, the paucity of UTUC samples constrained functional validation, precluding a comprehensive dissection of CAF function. Future studies should prioritize experimental validation and mechanistic exploration based on our multi-omics findings.

## Materials and methods

### All omics data sources

All patients signed informed consent forms for this study, and their clinical characteristics are recorded in Supplementary Table S1. All studies were approved by the Ethics Committee of The First People’s Hospital of Yunnan Province (KHLL2022-KY004). All methods were performed in accordance with relevant guidelines and regulations. We downloaded transcriptome data, mutation data, and clinical data for BUC (TCGA-BLCA) from The Cancer Genome Atlas (TCGA) database, ensuring that the omics data matched the clinical data. Additionally, we downloaded the corresponding transcriptome and clinical data for the GSE13507 cohort from the Gene Expression Omnibus (GEO) database, removing duplicates and retaining unique values for repeated gene probes (Xiao et al., 2022). The IMvigor210 cohort was downloaded to evaluate the immune response to the risk model, with a focus on urinary tract tumors (Mariathasan et al., 2018). The UTUC and BUC MICs were purchased from Outdo Biotech Company and included clinical information. The remaining tissue sequencing data were obtained from the First People’s Hospital of Yunnan Province, Kunming, China.

### snRNA-seq and scRNA-seq cohorts

Freshly resected tissues were divided into two groups: one for scRNA-seq and the other for conventional pathological diagnosis. The freshly resected tissues were rapidly frozen and stored at –80°C until nuclei extraction for snRNA-seq. No patients had received radiotherapy, chemotherapy, or other unique treatments prior to surgery. Adjacent non-tumor tissues were collected under the guidance of three chief physicians, at least 3 cm from the tumor margin, to ensure result accuracy. The scRNA-seq and snRNA-seq libraries were prepared using the 10× Genomics platform according to the manufacturer’s instructions. Briefly, following a series of experimental procedures, including cell counting and initial quality control, cDNA amplification, and gene expression library construction, high-quality sequencing data were obtained on the Illumina NovaSeq platform.

### Stereo-seq cohort

Approximately 1 cm of the resected tissue was selected, rinsed with phosphate-buffered saline, and immersed in pre-chilled tissue storage solution (Miltenyi Biotec). The tissue was then embedded in pre-chilled OCT (Sakura), and 3–4 consecutive 10-μm-thick frozen sections were cut for hematoxylin and eosin (H&E) staining and Stereo-seq library preparation. Brightfield images of the H&E specimens were obtained using a Motic microscope scanner for histopathological assessment. In brief, 100–200-μm-thick sections were cut from each OCT-embedded tissue, and total RNA was extracted using the RNeasy Mini Kit (Qiagen) following the manufacturer’s protocol. RNA integrity number (RIN) was determined using the 2100 Bioanalyzer (Agilent), ensuring that all tissues had the RIN ≥ 7.

The concentrations of the resulting PCR products were quantified using the Qubit™ dsDNA Assay Kit (Thermo Fisher). A total of 20 ng of DNA was then fragmented with in-house Tn5 transposase at 55°C for 10 min. The reactions were halted by adding 0.02% sodium dodecyl sulfate and gently mixing at 37°C for 5 min. The fragmented products were amplified under the following conditions: 25 ml of fragmentation product, 1× KAPA HiFi HotStart ReadyMix, 0.3 μM Stereo-seq-Library-F primer, and 0.3 μM Stereo-seq-Library-R primer in a total volume of 100 μl with nuclease-free H_2_O. The PCR protocol was: 1 cycle at 95°C for 5 min, followed by 13 cycles at 98°C for 20 sec, 58°C for 20 sec, and 72°C for 30 sec, and finally 1 cycle at 72°C for 5 min. PCR products were purified using AMPure XP Beads (ratios 0.63 and 0.153), used for DNA nanoball (DNB) generation, and subsequently sequenced on an MGI DNBSEQ-T7 sequencer.

### Processing of the snRNA-seq and scRNA-seq data

The Illumina platform was utilized to convert the original sequencing data into FASTQ files, and the human genome reference sequence (GRCh38) was used for comparison. The preliminary quality control and cell extraction from the original data were performed using the official analysis software CellRanger from 10× Genomics (https://support.10xgenomics.com/single-cell-gene-expression/software/overview/welcome).

Subsequently, the gene expression matrix of each cell was obtained. For subsequent analysis, we employed the ‘Seurat’ package (version 4.3.0) (Stuart et al., 2019). Based on the number of genes detected per cell/nucleus (300–5000), we retained 25% of the unique molecular identifier (UMI) count derived from internal mitochondria and considered the expression of each gene in at least three cells/nuclei, resulting in a total of 136042 high-quality cells/nuclei (for the sake of clarity, the entities mentioned below are collectively referred to as ‘cells’). To reduce data discretization, the data were normalized and then integrated and dimensionally reduced using the ‘harmony’ package (Korsunsky et al., 2019). We used the FindClusters function from the ‘Seurat’ package to identify the main cell clusters (resolution = 0.5) and selected the first 15 most important principal components for uniform manifold approximation and projection (UMAP) dimensionality reduction. The FindAllMarkers function in the ‘Seurat’ package was employed to identify genes significantly expressed in each cluster. Finally, we annotated 28 cell clusters, representing 7 major cell types.

### Processing of Stereo-seq data

FASTQ files were generated using the MGI DNBSEQ-T7 sequencer and aligned to the human genome GRCh38. The downloaded data were processed using the ‘Seurat’ package. The SCTransform function was used for data normalization. To address the challenge of low RNA-capture rates at high resolution, the raw spatial expression matrix was convolved into larger pseudo-points. SPOTlight software (version 1.0.1) (Elosua-Bayes et al., 2021) was then used to deconvolve cell type-specific profiles from paired scRNA-seq and snRNA-seq data, infer the cell type composition of each capture spot, and normalize the inferred compositions to assign and visualize the major cell types. Finally, the Monocle3 package (Cao et al., 2019) was used to plot the trajectory of cell differentiation.

### Differential gene enrichment analysis

The ‘FindMarkers’ function was utilized to identify differences in cell types between tumor and normal tissues, ensuring that the final outcomes had adjusted *P*-value < 0.05 and |log2FC| > 0.5. Subsequently, we conducted GO enrichment analysis separately for seven distinct cell types with the ‘clusterprofiler’ package (Supplementary Figure S1). Additionally, we extracted DEGs from the epithelial cells for GSVA. To investigate disparities in metabolic pathway activity between tumor and normal tissues, we employed the ‘scMetabolism’ package (Wu et al., 2022) with the following parameters: method=‘AUCell’ and metabolism.type=‘REACTOME’.

### CNV and NMF analyses in malignant epithelial cells

T cells were chosen as the control group, and the CNV level of epithelial cells was calculated using the InferCNV package (Tirosh et al., 2016) to infer their degree of malignancy. K-means unsupervised clustering was conducted on the results (*n* = 5) to observe the CNV scores of each cell subcluster, facilitating the preliminary classification of malignant epithelial cells into high-malignant and low-malignant. For further classification, the NMF package was applied to identify the internal characteristics of each UTUC patient. The characteristics were compared using hierarchical clustering, with the distance measure being 1 minus the Pearson correlation coefficient of all gene scores, and six modules were manually identified. The top 30 genes with the highest loading scores in each module (Supplementary Table S2) were extracted for GO enrichment analysis to identify their enrichment characteristics. Subsequently, Modules 1 and 2 were found to be enriched in high-malignant, Modules 3, 5, and 6 were enriched in low-malignant, while Module 4 exhibited both characteristics. Ultimately, malignant epithelial cells were defined as HMECs, MMECs, and LMECs. To observe intrinsic differences among UTUC patients, we employed the ‘GSVA’ package to score the top 30 genes with high expression in each module using the ssGSEA. Furthermore, we examined potential differences in pathways among subpopulations of malignant epithelial cells using GSVA.

### Malignant epithelial cell trajectory analysis

The ‘monocle’ package (Qiu et al., 2017) was utilized to identify changes in malignant epithelial cells. Low-quality genes were excluded, ensuring that each gene had an expression level of at least 0.1 and was expressed in a minimum of 10 cells. Subsequently, potential developmental features of malignant epithelial cells were examined using the BEAM.

### CAF subclusters

The fibroblasts were extracted from the tumor tissue, followed by re-clustering, resulting in the division of the cells into 10 distinct subclusters based on specific marker genes. Subsequently, a GO enrichment analysis was employed to observe pathway differences among the subclusters, and the Spearman correlation coefficients were calculated for each subcluster.

### Cell–cell communication

The relationship between malignant epithelial cells and CAFs was investigated using the Python package CellPhoneDB (version 3.1.0) (Efremova et al., 2020) for receptor–ligand pair analysis, employing default parameters. Receptor–ligand pairs with *P*-value <0.05 and expression level >1 were extracted, and the Spearman correlation coefficient was calculated between the cell subclusters exhibiting the most significant interaction.

### Characteristics of transcription factors in malignant epithelial cells

Transcription factor analysis was conducted utilizing the Python package pySCENIC (version 0.12.1) (Aibar et al., 2017). The co-expression module was constructed using GRNBoost2, and regulators were identified using the motif dataset (hg38_10kbp_up_10kbp_down_full_tx_v10_clust. genes_vs_motifs.rankings.feather). The input data were obtained from the count matrix of Seurat.

### Bulk samples of BUC

The Wilcoxon test was utilized to identify differential genes between BUC tissues and normal tissues in the TCGA-BLCA cohort. Genes with LogFC > 1, *P*-value < 0.05, and false discovery rate <0.05 were considered as differentially expressed. The obtained DEGs were intersected with the top 30 genes from each of the 6 NMF modules. The expression levels of the intersection genes in the TCGA-BLCA cohort were obtained, and univariate and multivariate Cox regression analyses were performed. Ultimately, five genes (LGALS3, BRINP3, PTPRR, XIST, and S100A16) that significantly influence the prognosis of UC were identified.

### Building CAF-related risk model

The expression levels of the top 30 marker genes of each CAF subcluster in the TCGA-BLCA cohort were extracted, and the ssGSEA scores were calculated for each subcluster. Survival analysis was performed using the ‘survival’ package. We determined the subcluster of CAFs that could potentially benefit from immunotherapy in the IMvigor210 cohort. Subsequently, we identified that myCAFs2 and myCAFs1 were associated with prognosis in UC patients. Univariate Cox regression analysis was conducted on the expression levels of the top 30 marker genes of both CAF subclusters to determine the factors related to overall survival in UC. The TCGA-BLCA cohort was randomly divided into two groups of patients with an equal number, and the training group underwent Lasso Cox regression screening, leading to the construction of a multivariate Cox regression model (Supplementary Table S3).

### Risk score

The risk score was defined as the sum of products of each gene’s expression level (Expi) and its corresponding risk coefficient (Coefi): Ɛ(Expi × Coefi). Based on the median risk score of the training group, the samples were categorized into high-risk and low-risk groups. We conducted further validation using risk models on the GSE13507 cohort and the IMvigor210 immunotherapy cohort.

### Significant mutation types of UTUC in BUC

The mutation status of the three most prominent mutation types of UTUC was analyzed in BUC using the ‘maftools’ R package. We examined the expression of mutation points, ligand pairs exhibiting the most significant interaction signals, each NMF module, immune checkpoints, risk genes, and co-expression characteristics in three bulk cohorts.

### Multiplex immunofluorescence on tissue microarrays

We performed immunofluorescence imaging using previously reported procedures (Toth and Mezey, 2007; Stack et al., 2014). The Opal 6 Plex Detection Kit for Whole Slide Imaging (NEL871001KT, PerkinElmer) was employed. Briefly, the tissue microarrays were baked in an oven at 65°C for 1 h and then deparaffinized in xylene (3 times, 10 min each). The sections were rehydrated through a graded series of ethanol solutions: 100% (1 time, 10 min), 95% (1 time, 10 min), and 70% (rinse). After rehydration, the slides were briefly rinsed in distilled water and fixed in 10% neutral buffered formalin for 20 min. Next, the microarrays were blocked for 10 min and incubated for 1 h with one of the following primary antibodies for immunofluorescence staining: COL1A1 (1:100, ARG21965, Arigo), FN1 (1:100, ab32419, Abcam), or CALM1 (1:100, MA3-917, Thermo Fisher). After washing with Tris-buffered saline with 0.1% Tween 20 (TBST), the sections were incubated for 10 min with secondary antibodies (1X Opal Anti-Ms + Rb HRP and Anti-Goat HRP, Akoya Biosciences). The slides were then removed, and excess TBST was discarded. The slides were placed in a humid chamber, and Opal TSA-DIG working solution was added to cover the entire tissue, incubating at room temperature for 10 min. Following incubation, the slides were washed three times with TBST on a shaker, 2 min each time, to remove the secondary antibody. Antibody complexes were removed using microwave treatment. Subsequent markers were counterstained, and these steps were repeated until all markers had been evaluated. The slides were then counterstained with 4′,6-diamidino-2-phenylindole (DAPI) for 5 min and mounted (ProLong® Diamond Antifade Mountant, Thermo Fisher).

### Statistical analysis

Statistical analysis and graphing for this study were conducted using R software (version 4.2.2), ImageJ (version 1.54f), Python (version 3.7.16), Cytoscape (version 3.10.0), and GraphPad Prism 9.5. Unless specified, differences are considered significant only when *P* < 0.05. In this context, NS (not significant), *P < 0.05, **P < 0.01, ***P < 0.001, and ****P < 0.0001. Student’s *t*-test, Wilcoxon rank-sum test, and analysis of variance (ANOVA) (for three or more groups) were used for continuous variables. Paired comparisons were conducted using paired *t*-tests. Any additional methods are described in the corresponding sections of the manuscript.

## Supplementary Material

mjaf032_Supplemental_Files

## Notes

**Author contributions:** Y.X. and C.Z. designed the study; Y.X., J.Y., M.S., Y.L., and J. Li performed experiments; Q.L., L.K., F.H., Y.Y., and C.Z. analyzed the data; C.Z. and Y.X. wrote the manuscript. All authors read and approved the final manuscript.
