# Supplementary material for: The tumor-associated fibroblasts regulate urothelial carcinoma progression
Source: J Mol Cell Biol. 2025 Sep 19;17(9):mjaf032. doi: 10.1093/jmcb/mjaf032 (PMC13032880; doi:10.1093/jmcb/mjaf032)
Supplement: mjaf032_Supplemental_Files [file mjaf032_supplemental_files.zip › JMCB-2025-0330.R2_Supplementary Figures.docx]

**Supplementary Figures**


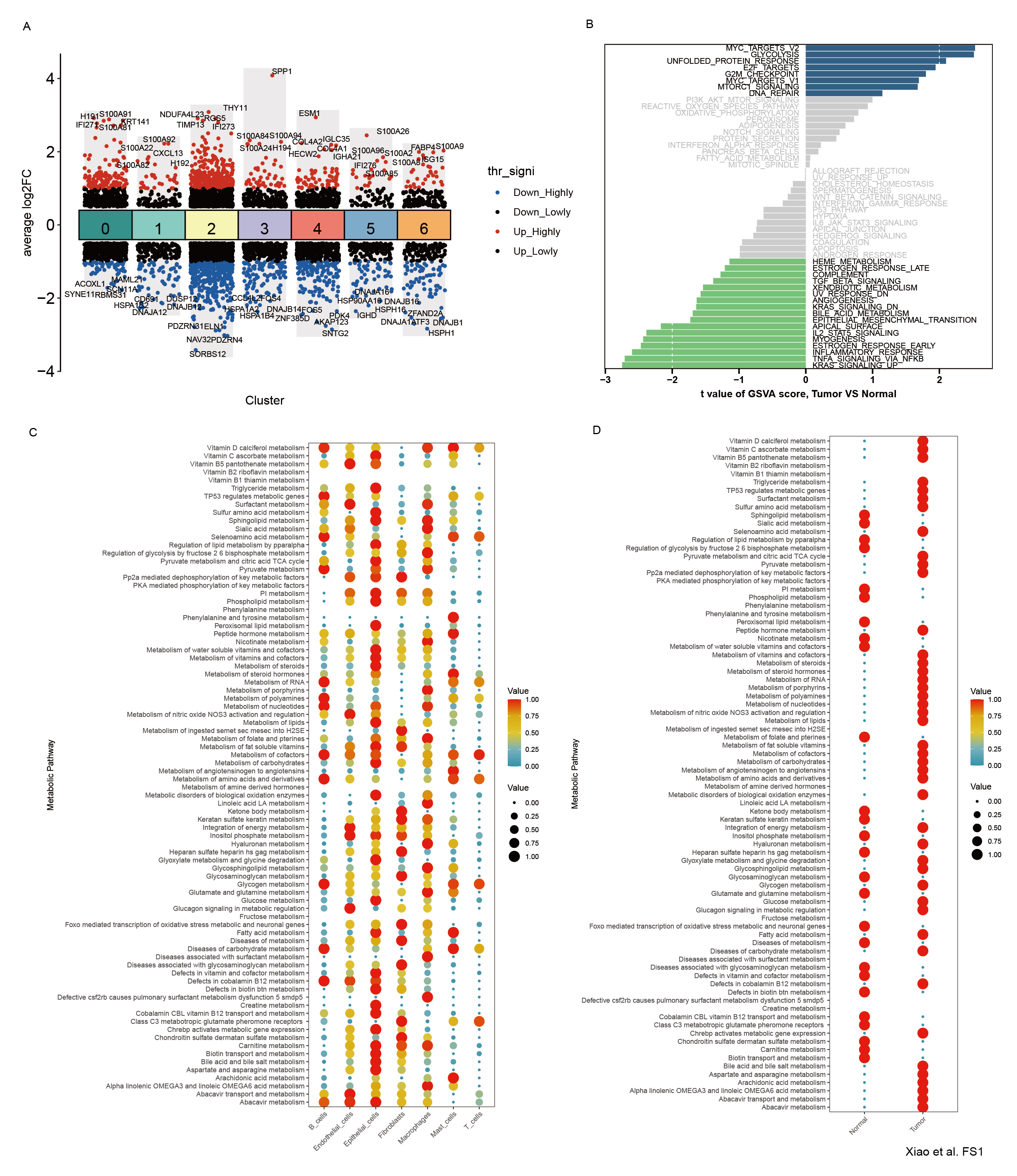


**Fig. S1. Analysis of differences between UTUC and para-cancer tissues.**

(**A**) Differential analysis was performed on 7 cell types between tumor and normal tissue, with numbers 0 to 6 assigned to represent epithelial cells, T cells, macrophages, fibroblasts, endothelial cells, B cells, and smooth muscle cells, and show the top five DEGs. (**B**) The GSVA scores of tumors and normal epithelial cells were compared, and |t-value| > 1 indicated a significant difference. In the representation, the upper half corresponds to tumor epithelium, while the lower half represents normal epithelium. (**C** and **D**) Bubble plot of metabolic difference between tumor and normal tissues.


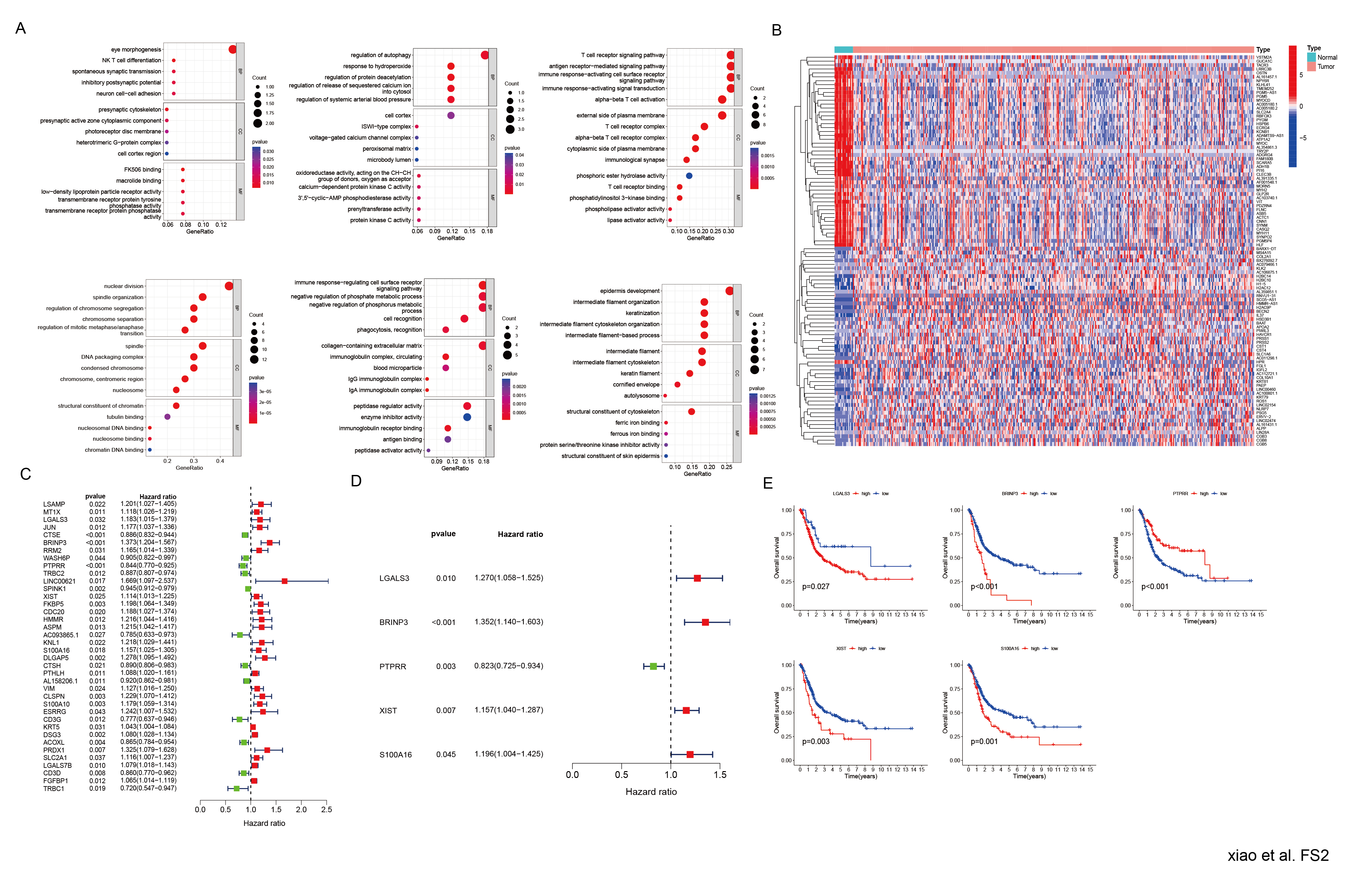


**Fig. S2.** **Prognostic characterization of NMF module genes.**

(**A**) GO enrichment analysis of NMF modules. (**B**) Heatmap showing differences between normal and tumor tissues in the TCGA-BLCA cohort. (**C** and **D**) Univariate and multivariate COX regression analyses of intersection gene. (**E**) Kaplan–Meier curves showing the survival of five genes with different expression patterns in the TCGA-BLCA cohort.


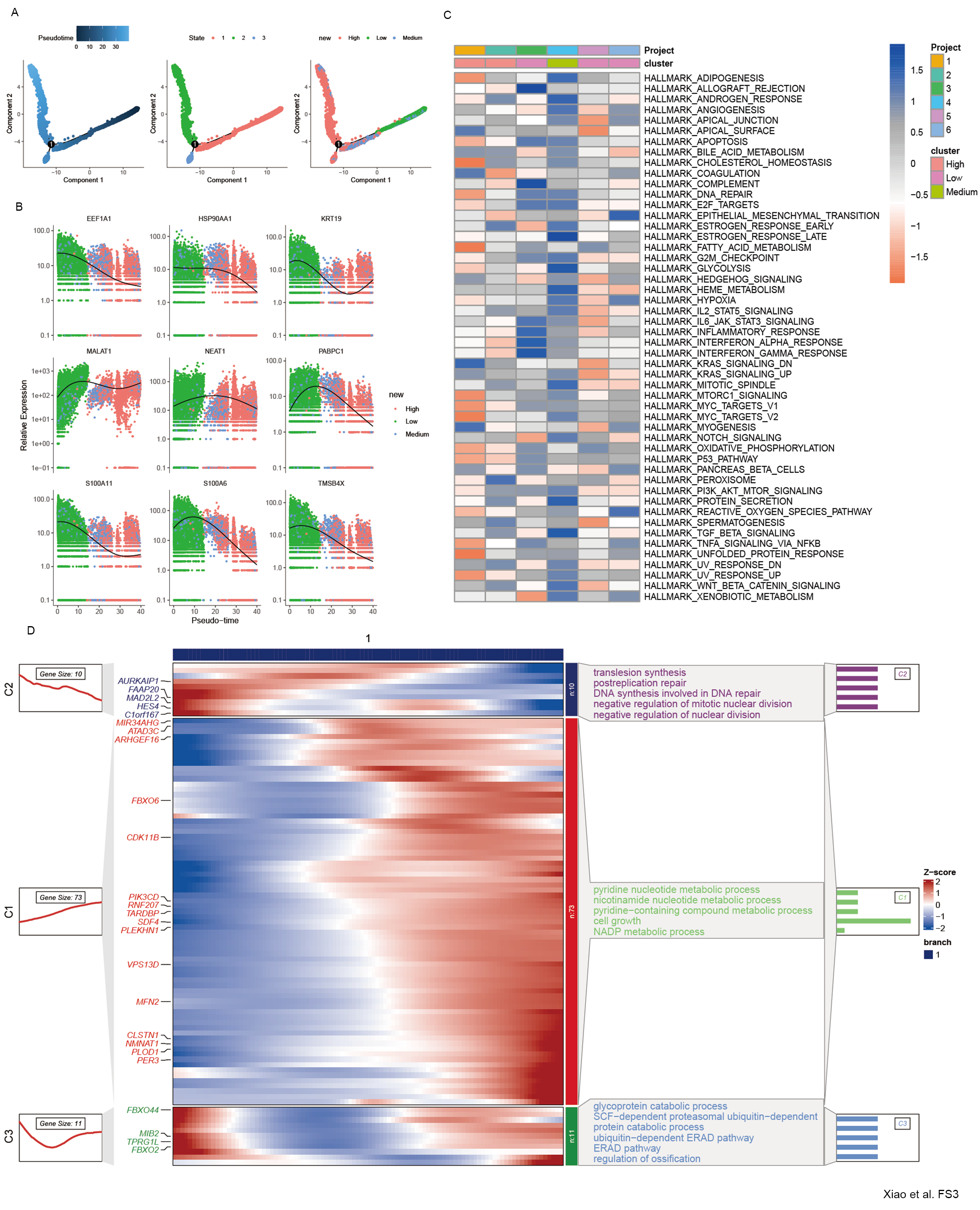


**Fig. S3. Pseudotime trajectory characteristics of tumor epithelial cells.**

(**A**) The differentiation trajectory of UTUC epithelial cell is illustrated from left to right, depicting the pseudotime, state composition, and cell types. (**B**) The dynamics of the top 9 genes in UTUC epithelial cells are analyzed, with the horizontal axis denoting pseudotime and the vertical axis indicating gene expression levels. (**C**) The GSVA pathway characteristics of different epithelial cells and NMF modules are investigated, higher expression levels in most pathways for MMECs. (**D**) The BEAM analysis was used to observe the change characteristics of epithelial cell branches. The alterations in the top 100 genes were displayed and the top 25 genes were labeled. Notably, the expression level of the C1 gene exhibited a gradual increase, while the C2 gene showed a gradual decrease, and the C3 gene displayed fluctuation. Furthermore, the most significant GO pathway of the 3 states of epithelial cells is marked.


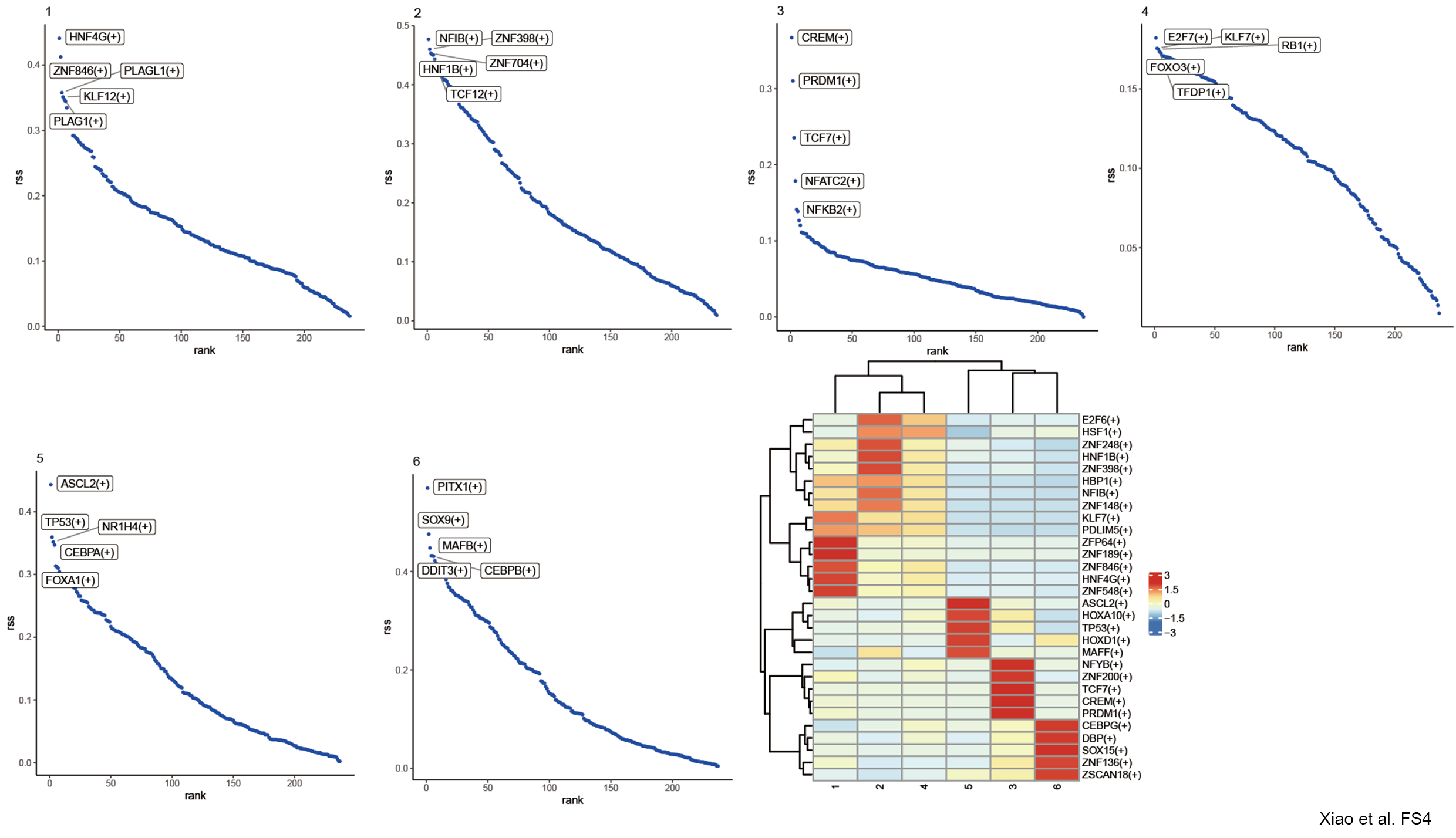


**Fig. S4. TFs characteristics of different NMF modules.**


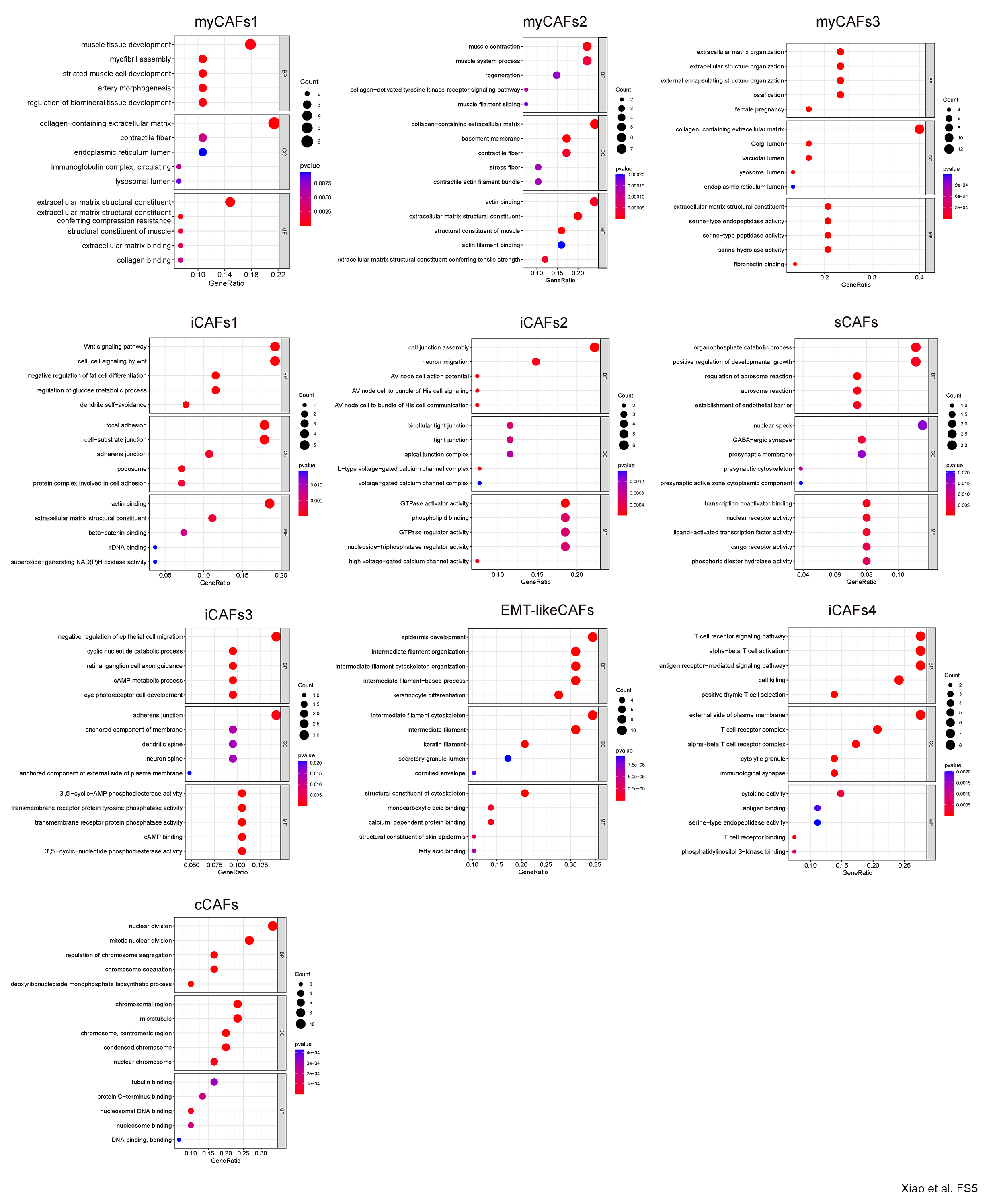


**Fig. S5. GO enrichment analysis of CAF subclusters.**


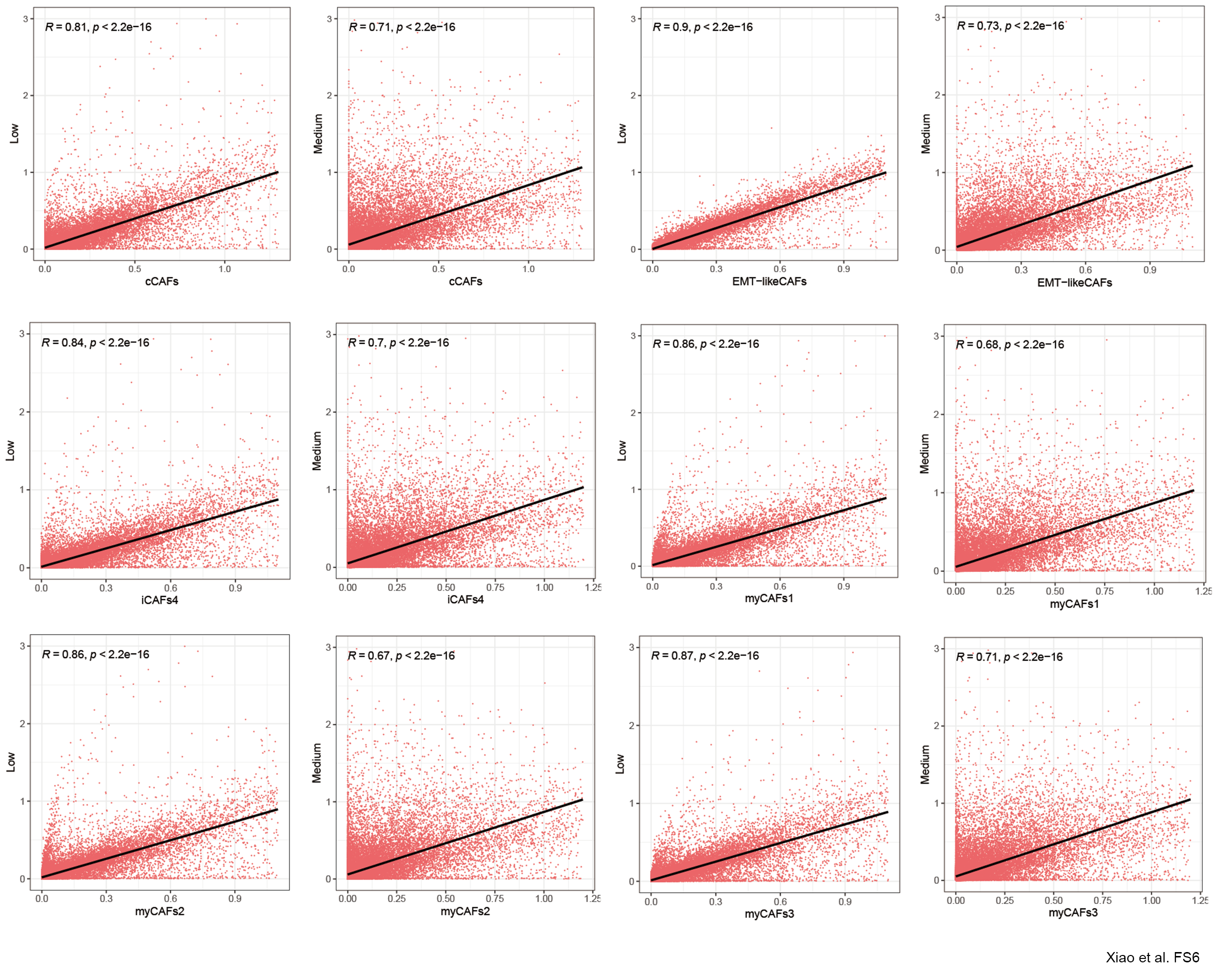


**Fig. S6. The scatter plot of correlation between CAFs and epithelial cells showed a positive correlation.**


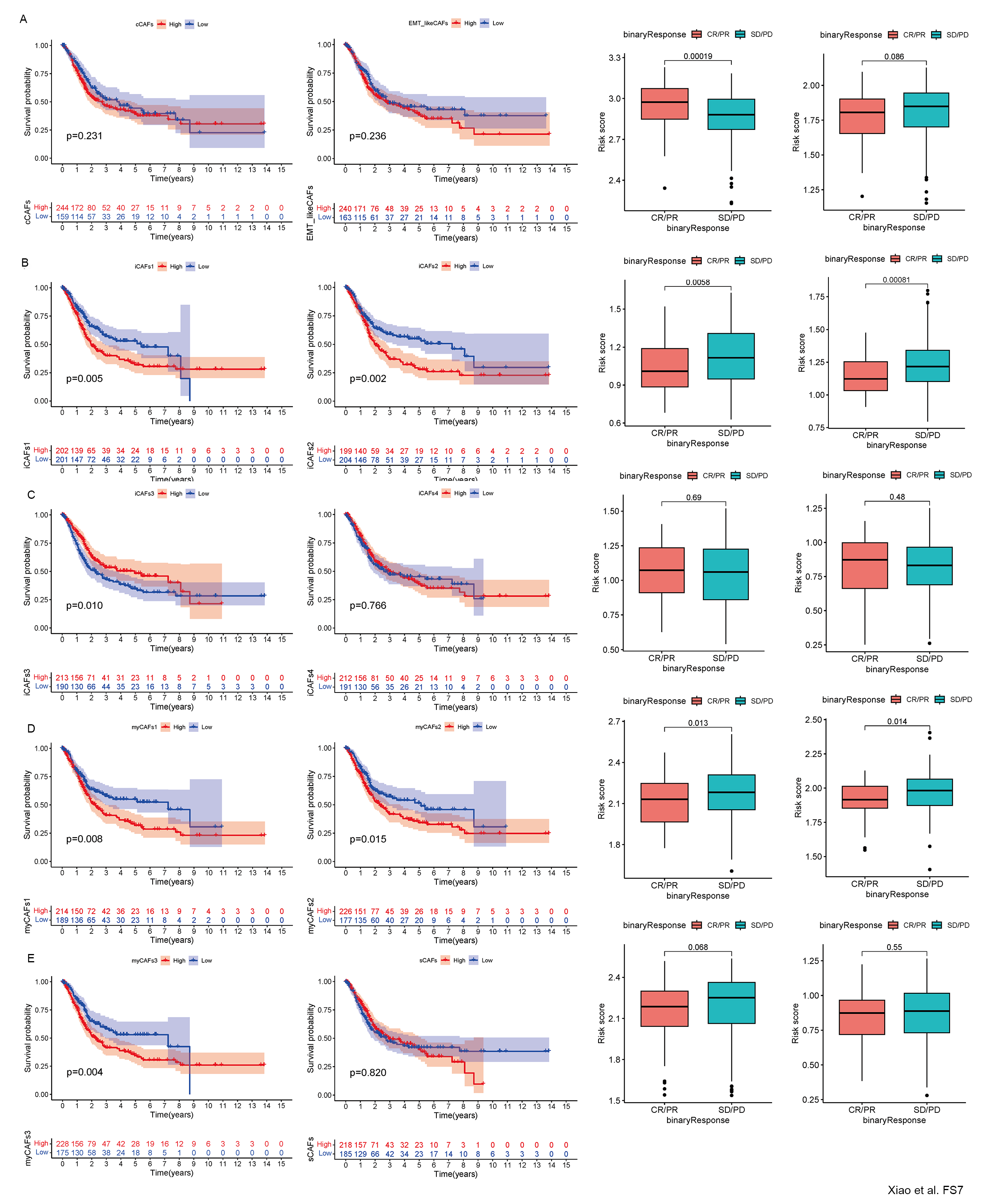


**Fig. S7. Prognostic and immunotherapeutic characterization of CAFs subclusters.**

Kaplan-Meier curves and immune response differences for CAF subclusters with different expression models. The results for myCAF1 and myCAF2 were the most favorable.


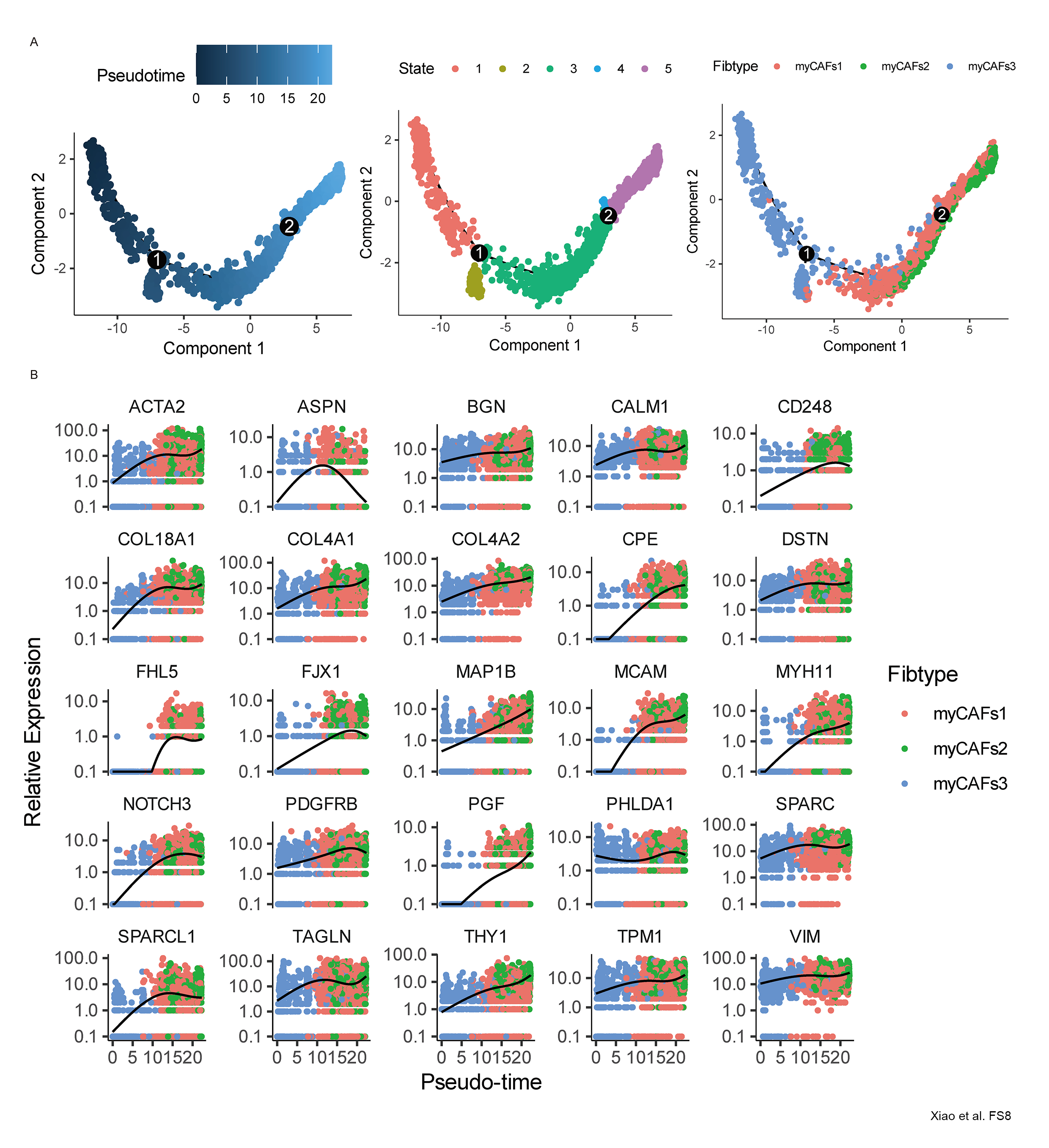


**Fig. S8. Pseudotime trajectory characteristics of myCAFs.**

(**A**) Pseudotime characteristics of myCAF subclusters, with myCAF1 and myCAF2 in the terminal stage of development. (**B**) Developmental trajectory of prognostic-related genes shown in Figure 8A.


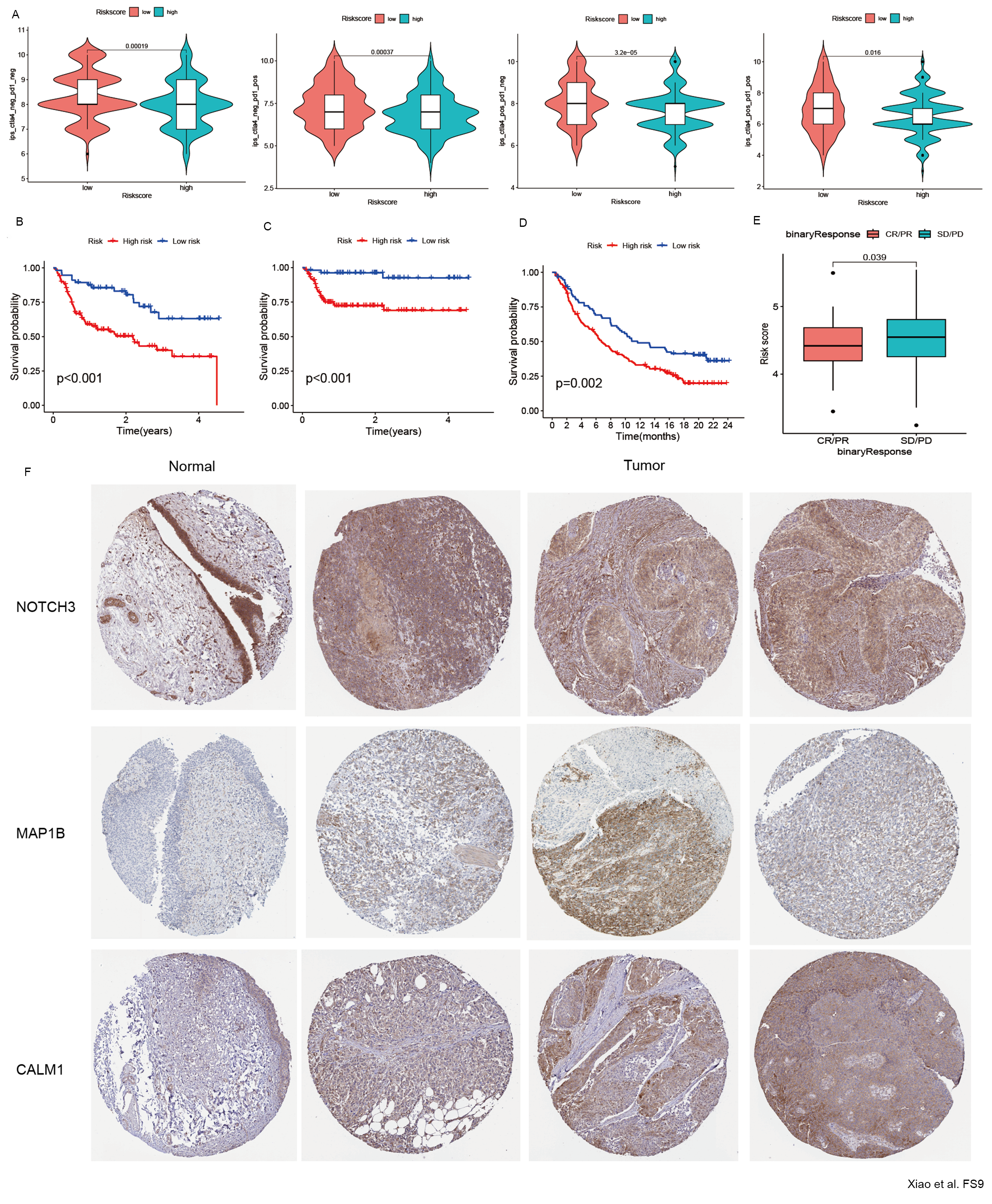


**Fig. S9. Multi-Cohort Validation of Risk Model Reliability.**

(**A**) The response to immunotherapy varies between the high-risk group and the low-risk group, with the low-risk group showing a greater benefit from the treatment. (**B**–**D**) In the external validation cohort, the survival analysis of the risk model revealed significant differences between the high-risk and low-risk groups. From left to right, the analysis represents the overall survival of the GSE13507 cohort, the progression-free survival, and the overall survival of the IMvigor210 cohort. (**E**) Difference in immunotherapy response were observed between high-risk and low-risk groups in IMvigor210 cohort, with low-risk groups displaying more pronounced immune responses. (**F**) Immunohistochemical validation of risk model genes.


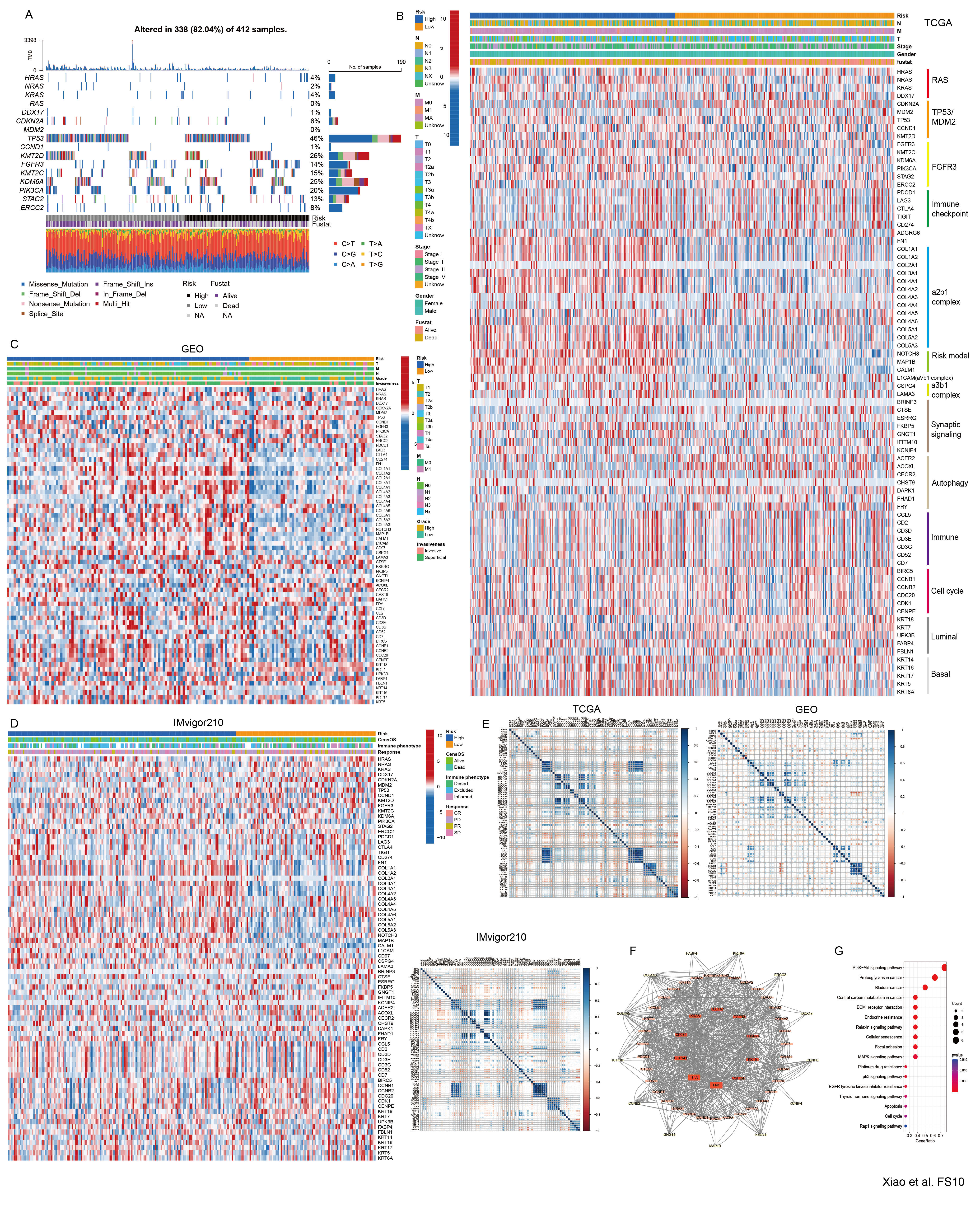


**Fig. S10. Gene sets have consistent expression patterns across cohorts.**

(**A**) The significant mutational characteristics of UTUC is different from BUC. (**B**–**D**) Expression characteristics of different gene sets in three cohorts, showing consistent expression patterns across the cohorts. (**E**) Correlated expression of each gene in different gene sets across three cohorts, showing similar characteristics. (**F**) Protein interaction patterns of genes in different gene sets (https://cn.string-db.org/), evaluated using Cytoscape_v3.10.0 to assess Betweenness Centrality, arranged from inside to outside in descending order. (**G**) Significant enrichment pathways for the top 10 genes.


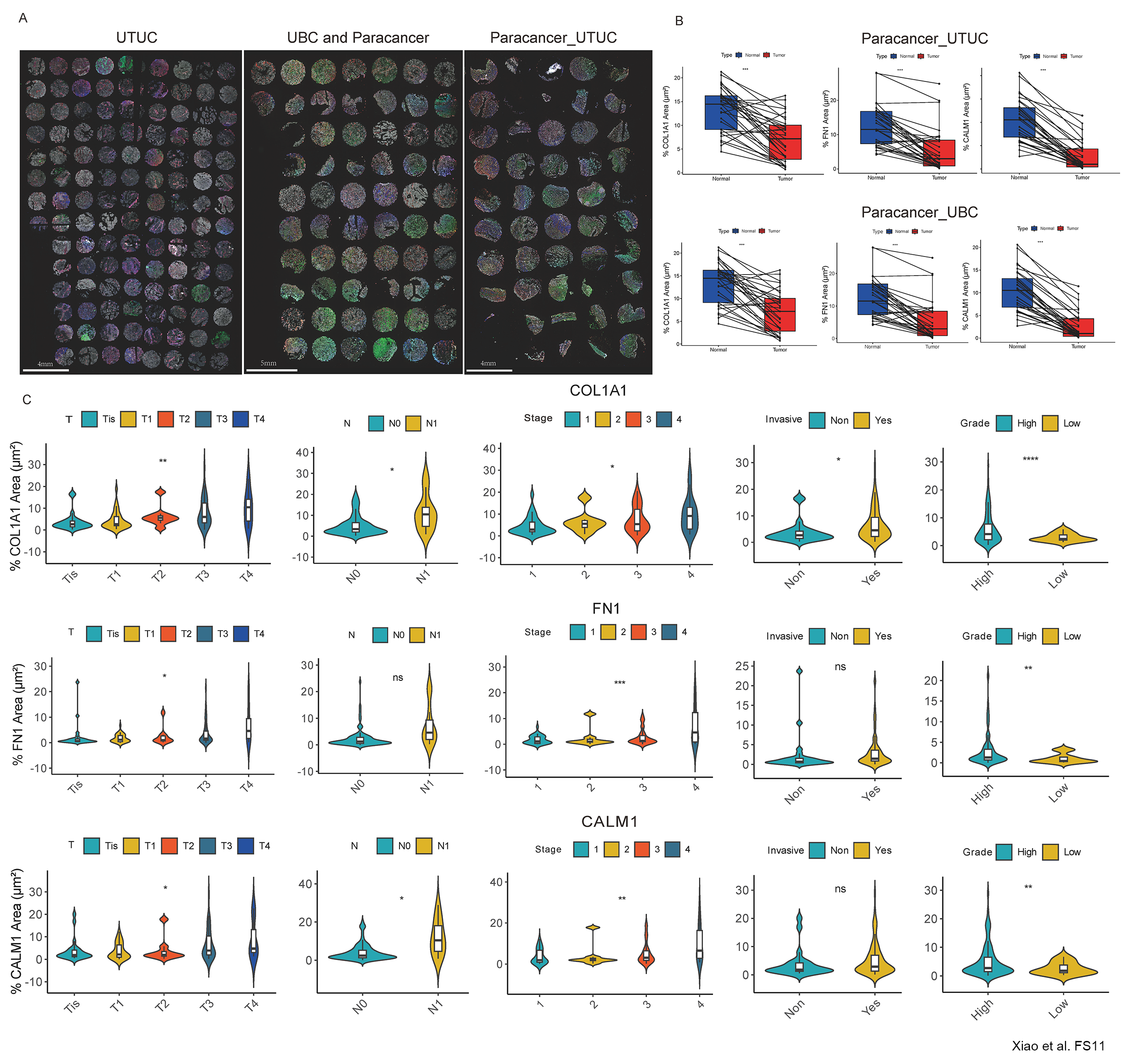


**Fig. S11. FN1, COL1A1 and CALM1 predict clinicopathologic features of both UTUC and BUC.**

(**A**) Panoramic images of three groups of immunofluorescence chips, in which BUC was odd rows and paired para-cancerous tissue was even rows. (**B**) Paired difference analysis of UTUC and BUC with corresponding para-cancerous tissues. (**C**) High expression of FN1, COL1A1 and CALM1 in UTUC has worse clinicopathological features.


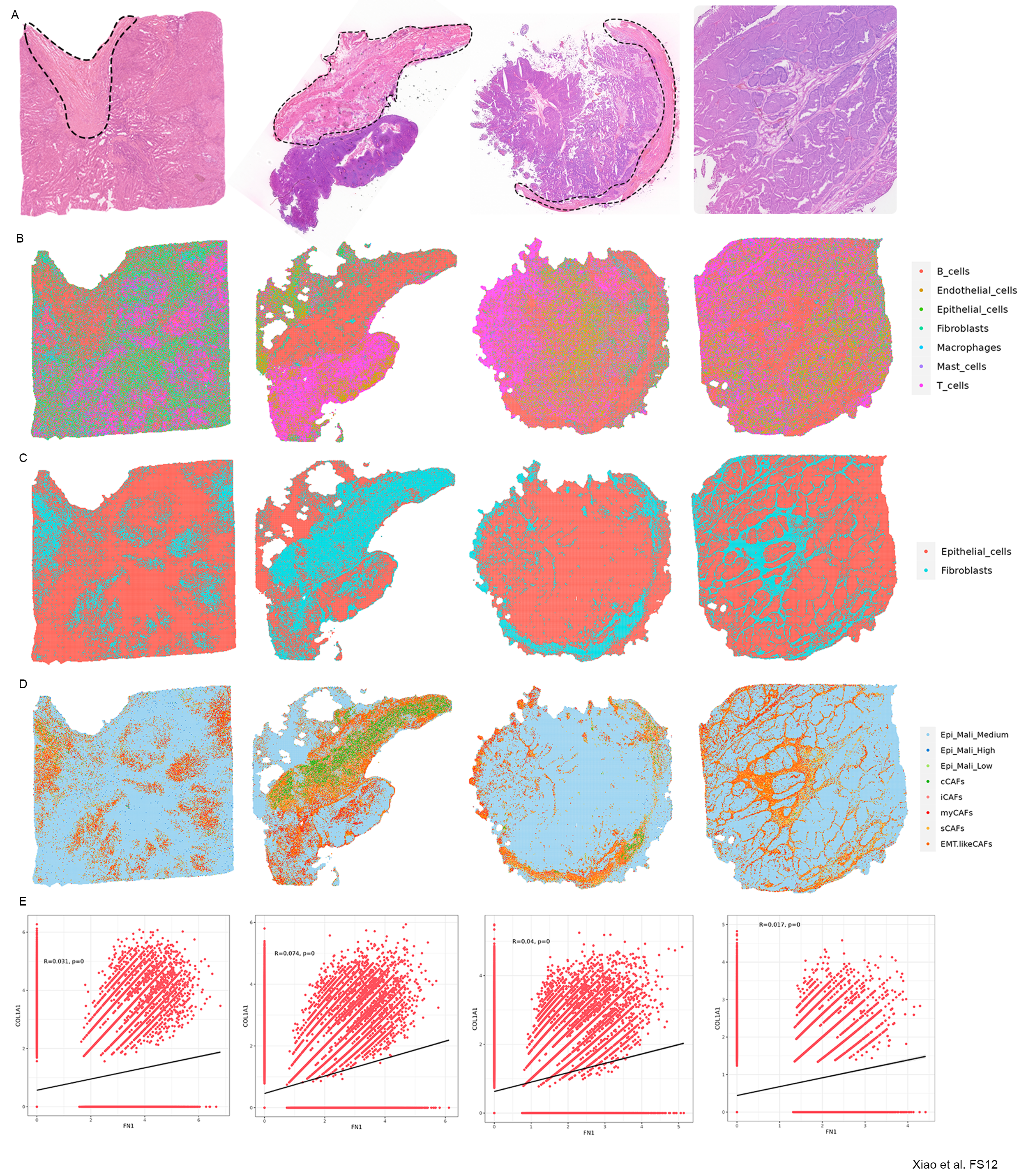


**Fig. S12. Spatial transcriptome validation of a positive correlation between FN1 and COL1A1 expression.**

(**A**–**D**) Spatial transcriptome characteristics of 4 UTUC samples. The dotted line box represents the tumor margin area. (**E**) Expression correlation of FN1 and COL1A1 in different samples. The results showed a positive correlation.


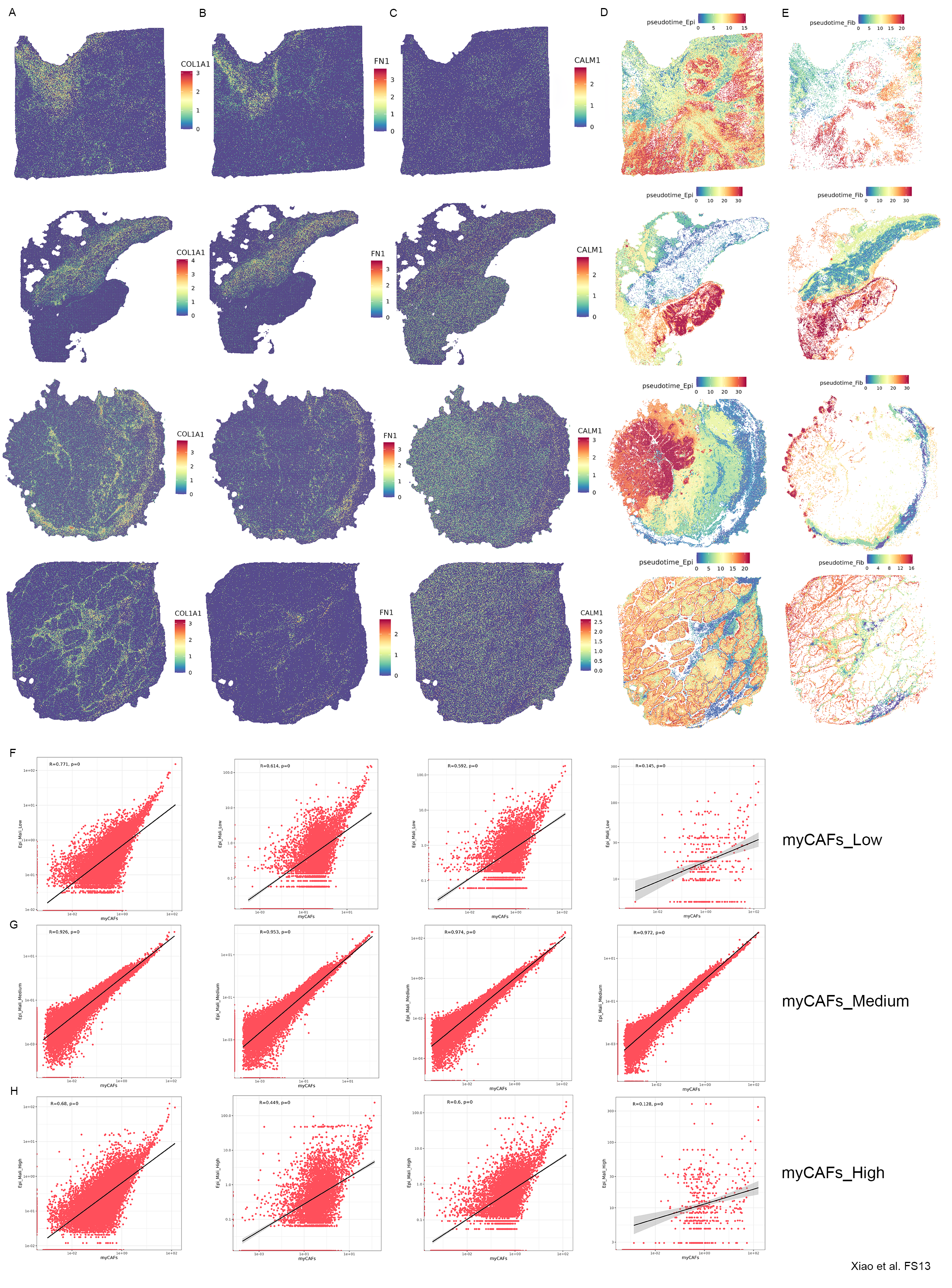


**Fig. S13. Spatial transcriptome validation of positive correlation between myCAFs and epithelial cells.** (**A**–**C**) Expression characteristics of FN1, COL1A1, and CALM1 in the UTUC spatial transcriptome, with spatial co-localization of FN1 and COL1A1. The chip size is 1x1 cm. (**D** and **E**) Spatial pseudotime trajectory characteristics of epithelial cells and CAFs, showing gradual differentiation from the tumor margin inward. (**F**–**H**) Correlated expression of myCAFs with the three groups of malignant epithelial cells, showing a positive correlation.


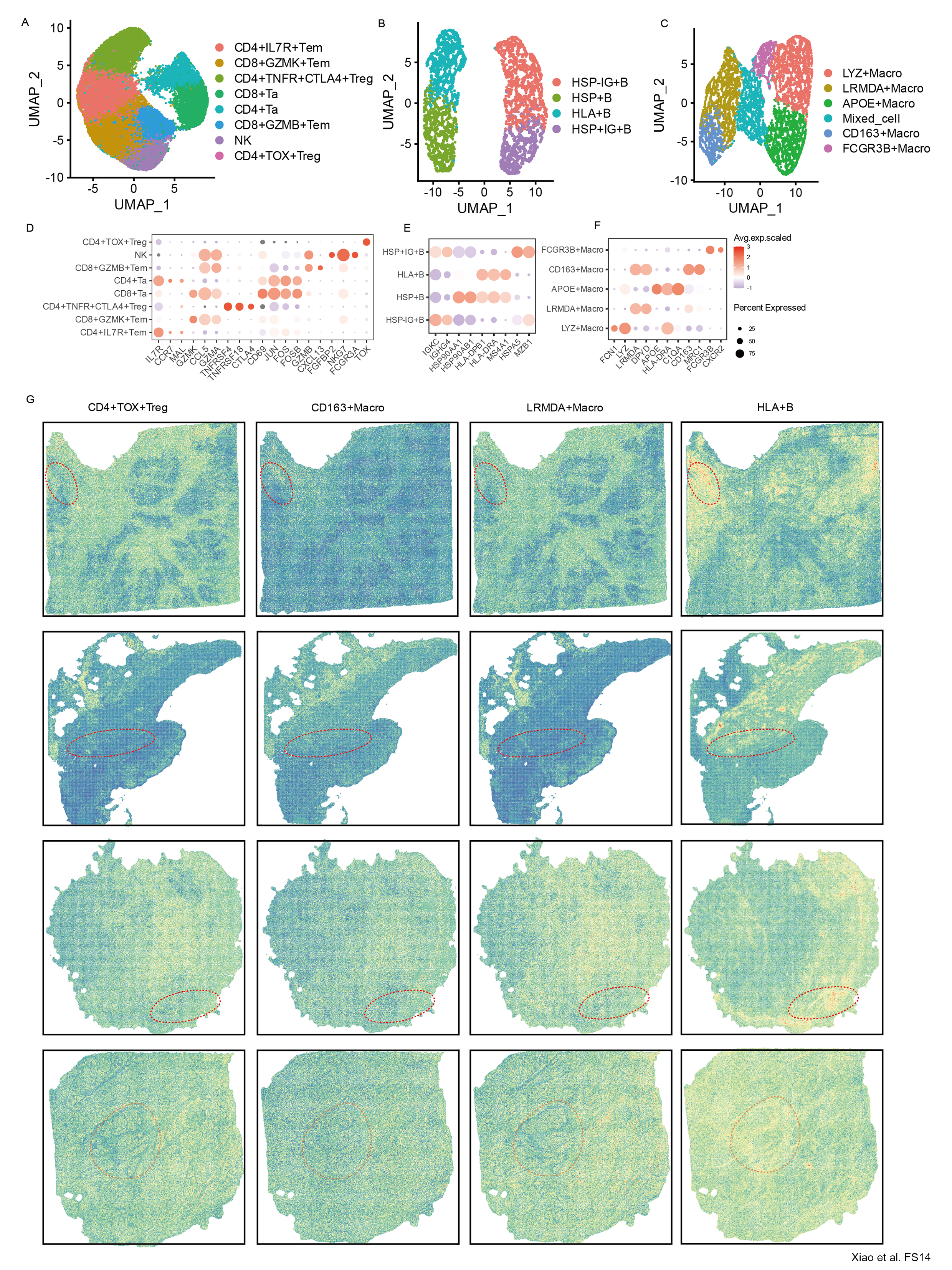


**Fig. S14. The Immune Characteristics of the UTUC.**

(**A**–**C**) Subtype clustering of T cells, B cells, and macrophages, respectively. (**D**–**F**) Bubble plots showing subtype-specific markers for T cells, B cells, and macrophages, respectively. (**G**) Spatial distribution in the four UTUC samples showing the localization of CD4+TOX+Treg, LRMDA+Macro, CD163+Macro and HLA+B.
